# Genus-Specific Interactions of Bacterial Chromosome Segregation Machinery Are Critical for Their Function

**DOI:** 10.3389/fmicb.2022.928139

**Published:** 2022-07-06

**Authors:** Monika Pióro, Izabela Matusiak, Adam Gawek, Tomasz Łebkowski, Patrycja Jaroszek, Matthieu Bergé, Kati Böhm, Judith Armitage, Patrick H. Viollier, Marc Bramkamp, Dagmara Jakimowicz

**Affiliations:** ^1^Faculty of Biotechnology, Department of Molecular Microbiology, University of Wrocław, Wrocław, Poland; ^2^Department of Microbiology and Molecular Medicine, University of Geneva, Geneva, Switzerland; ^3^Faculty of Biology, Ludwig-Maximilians-Universität München, Planegg-Martinsried, Germany; ^4^Department of Biochemistry, University of Oxford, Oxford,United Kingdom; ^5^Institute of General Microbiology, Kiel University, Kiel, Germany

**Keywords:** segregation proteins, ParA, ParB, DivIVA, polar recruitment

## Abstract

Most bacteria use the ParABS system to segregate their newly replicated chromosomes. The two protein components of this system from various bacterial species share their biochemical properties: ParB is a CTPase that binds specific centromere-like *parS* sequences to assemble a nucleoprotein complex, while the ParA ATPase forms a dimer that binds DNA non-specifically and interacts with ParB complexes. The ParA-ParB interaction incites the movement of ParB complexes toward the opposite cell poles. However, apart from their function in chromosome segregation, both ParAB may engage in genus-specific interactions with other protein partners. One such example is the polar-growth controlling protein DivIVA in Actinomycetota, which binds ParA in *Mycobacteria* while interacts with ParB in *Corynebacteria*. Here, we used heterologous hosts to investigate whether the interactions between DivIVA and ParA or ParB are maintained across phylogenic classes. Specifically, we examined interactions of proteins from four bacterial species, two belonging to the Gram positive Actinomycetota phylum and two belonging to the Gram-negative Pseudomonadota. We show that while the interactions between ParA and ParB are preserved for closely related orthologs, the interactions with polarly localised protein partners are not conferred by orthologous ParABs. Moreover, we demonstrate that heterologous ParA cannot substitute for endogenous ParA, despite their high sequence similarity. Therefore, we conclude that ParA orthologs are fine-tuned to interact with their partners, especially their interactions with polarly localised proteins are adjusted to particular bacterial species demands.

## Introduction

Although most bacteria use the same machinery to replicate and segregate their chromosomes, they employ different strategies to coordinate their cell cycle. Because of adaptations to various environmental niches, bacterial species differ significantly with respect to cell shape, generation time, mode of growth, and metabolic diversity. The demands of diverse cell cycles or modes of growth require cell cycle checkpoints that depend on specific protein–protein interactions. In the majority of bacterial species, cell division by binary fission directly follows chromosome duplication and segregation (Thanbichler, 2010; Reyes-Lamothe et al., 2012). Chromosome replication is initiated at the origin of replication (*oriC*) region. Shortly after duplication, daughter *oriC*s are actively separated and moved towards the future daughter cell(s), while the rest of the chromosome still undergoes replication (Webb et al., 1997; Lemon and Grossman, 2000; Viollier et al., 2004; Bates and Kleckner, 2005; Reyes-Lamothe et al., 2008; Trojanowski et al., 2018). At the same time, the bacterial cells grow, which in the case of rod-shaped bacteria results from the extension of either the lateral cell wall or cell poles. The position of *oriC*s in new-born cells is precisely controlled, but it may differ even among closely related bacteria, such as *Corynebacteria* and *Mycobacteria* (Donovan et al., 2010; Trojanowski et al., 2015; Böhm et al., 2017). In most bacteria (with the exception of gammaproteobacteria), the segregation and subsequent positioning of *oriC* depends on chromosome segregation proteins, namely, ParA and ParB. Interestingly, these proteins are also involved in interactions that allow the coordination of cell cycle processes (Pióro and Jakimowicz, 2020).

Segregation proteins share their biochemical properties in all studied bacterial species, with ParA being a P–loop ATPase and ParB a CTPase which, thanks to the HTH domain, binds to *parS* DNA sequences. In almost 70% of the studied bacterial chromosomes, *parS* sequences are located in the vicinity of *oriC*, however, their number and exact positioning differ among bacteria (Jakimowicz et al., 2007; Livny et al., 2007; Toro et al., 2008; Dubarry et al., 2019; Böhm et al., 2020). ParB interactions with *parS* are followed by CTP-dependent ParB spreading at neighbouring DNA. These interactions lead to the formation of higher-order nucleoprotein complexes (segrosomes; Osorio-Valeriano et al., 2019; Soh et al., 2019; Jalal et al., 2020; Babl et al., 2022), which are actively moved by ParA. Upon binding ATP, ParA forms a dimer that non-specifically interacts with DNA. Nucleoprotein complexes formed by ParB stimulate the ATPase activity of ParA and trigger its release from DNA, resulting in a concentration gradient of nucleoid-bound ParA. The ParB complexes are moved towards higher concentrations of DNA-bound ParA, leading to separation of replicated *oriC* regions (Leonard et al., 2004, 2005; Livny et al., 2007; Lim et al., 2014; Hu et al., 2017).

Interestingly, the positioning of *oriC* regions and segrosomes, as well as the pattern of their segregation, differs among the studied bacterial species. In a free living curved alphaproteobacterium *Caulobacter crescentus*, cell division generates two cell types, flagellated and stalked with *oriCs*, located at the old cell pole. Upon initiation of replication (which takes place in stalked cell), one of the *oriCs* and segrosomes is moved towards the opposite cell pole (Jensen et al., 2001; Toro et al., 2008). Closely related *Rhodobacter sphaeroides* possesses two chromosomes, divides symmetrically and positions both *oriC* in distinct manner, with *oriC*I and ParB1 located similarly to *C. crescentus* (Dubarry et al., 2019). However, polarly extending cells of two related Actinomycetota species, i.e., *Mycobacterium smegmatis* and *Corynebacterium glutamicum*, differ in chromosome arrangement. In fast-growing club-shaped *C. glutamicum*, overlapping rounds of chromosome replication result in multiple copies of chromosomes in the cells, and in newborn cells, *oriCs* are localised at both cell poles (Böhm et al., 2017). Contrary, in slow-growing *M. smegmatis oriC* of a single chromosome is positioned at some distance from the pole, and upon initiation of replication, both *oriCs* are moved in a partially asymmetrical manner (Ginda et al., 2017). This indicates the existence of different cell cycle checkpoints even in closely related bacterial species.

The specific positioning of *oriC* is usually dependent on ParA or ParB interactions. Anchoring *oriC* at the cell poles is mediated in *C. crescentus* due to the interaction of ParB complexes with cell pole scaffold PopZ protein (Holmes et al., 2016). PopZ interacts at least eight other proteins and also ParA-PopZ interactions contribute to the chromosome segregation (Ptacin et al., 2014). Interestingly while in *C. glutamicum*, ParB interacts with the polar growth determinant DivIVA (Donovan et al., 2012), in *M. smegmatis*, ParA instead of ParB was shown to be a DivIVA interaction partner and this interaction facilitates segrosome separation and affects cell elongation (Ginda et al., 2013; Pióro et al., 2019). In *C. crescentus*, ParA also interacts with TipN, the protein localised at the new cell pole, and this interaction is required for proper chromosome segregation (Schofield et al., 2010). Moreover, the ParB protein controls cell division thanks to the interaction with the regulator of FtsZ polymerisation, MipZ protein in *C. crescentus* and *R. sphaeroides* or directly with FtsZ in *C. glutamicum* (Thanbichler and Shapiro, 2006; Donovan et al., 2010; Dubarry et al., 2019). Finally, by recruiting the structural maintenance of the chromosome (SMC) complex, ParB protein coordinates chromosome segregation with chromosome compaction (Gruber and Errington, 2009; Sullivan et al., 2009; Minnen et al., 2011; Chan et al., 2020).

Due to its numerous interactions, the ParABS system plays a role in the control of the bacterial cell cycle, and its varied functions are manifested by a variety of phenotypes that result from *parAB* deletion. In some bacteria, including *C. crescentus*, the *parAB* genes are essential for cell survival (Mohl and Gober, 1997), but in many other bacterial species, their deletion is possible but causes a range of chromosome defects from mild to more severe (Jakimowicz et al., 2007; Donovan et al., 2010; Ginda et al., 2013). Overproduction of ParA in *C. crescentus*, *M. smegmatis*, and *C. glutamicum* also impacts culture growth, altering cell length and impairing chromosome segregation, but usually to a lesser extent than deletion (Mohl and Gober, 1997; Donovan et al., 2010; Ginda et al., 2013). Thus, ParA and ParB proteins not only execute chromosome segregation but also indirectly influence cell elongation.

Comparative bioinformatics analysis revealed that ParABS systems are very conserved among a broad range of studied bacteria (Livny et al., 2007). On the other hand, recent studies show that components of the ParABS system take part in many different cellular processes, which indicates that they acquired new functions during evolution. The genus-specific interactions raise the question of compatibility of ParAB systems among different bacterial species. Here, we compared the heterologous interactions of ParAB proteins from four different bacterial species, two Gram-positive and two Gram-negative. Next, we examined ParA functionality in heterologous systems using *M. smegmatis* and *C. crescentus* as the host for overexpression of closely and distantly related homologues. Finally, we focused on most closely related actinobacterial ParA proteins and we tested their combability in *C. glutamicum* and *M. smegmatis* using complementation assays.

## Materials and Methods

### Cloning and Construct Preparation

DNA manipulations were performed using standard protocols (Sambrook and Russell, 2001). Reagents and enzymes were supplied by New England Biolabs (NEB), Merck, Roth and Thermo-Scientific. Oligonucleotides were synthesized by Merck, Genomed, and Microsynth, and sequencing was performed by Microsynth. The genetic construct preparation and *M. smegmatis*, *C. glutamicum*, and *C. crescentus* modifications are described in detail in the Supplementary Material.

### *Escherichia coli* Growth Conditions

*Escherichia coli* strains were grown in lysogeny broth (LB) medium at 37°C [DH5α, BL21(DE3)] or 30°C (BTH101). Culture conditions, antibiotic concentrations, and transformation protocols followed standard procedures (Sambrook and Russell, 2001).

### BTH Analysis and Fluorescence Microscopy Analysis of *Escherichia coli*

Bacterial two-hybrid (BTH) interaction studies were performed as previously described (Karimova et al., 1998). To analyse the interaction between the studied proteins in the BTH system, pUT18C and pKT25 derivatives with analysed genes were transformed into *E. coli* BTH101 and plated on LB containing 0.004% X-gal, 50 μg/ml kanamycin, 100 μg/ml ampicillin, and 0.5 mM isopropyl-β-D-1-thiogalactopyranoside (IPTG). After 2 days of incubation at 30°C, the selected representative colonies of each transformation were plated together on LB containing X-gal, kanamycin, ampicillin, and IPTG.

For assays of ParAs and DivIVA colocalisation in *E. coli*, BL21 (DE3) cells containing pJP108 (Ptacin et al., 2010), pJP108*divIVA*_Ms_ (Pióro et al., 2019), or pCD74 containing *divIVA_Cg_-mcherry* gene and pACYCDuet-1 vector derivatives containing *egfp-parA* genes were cultured to log phase in the presence of ampicillin and chloramphenicol. The expression of the *divIVA_Cg_*-*mcherry* and *egfp-parA* genes was induced by the addition of 0.1 mM IPTG, while the expression of *ics-mcherry* and *mcherry-divIVA_Ms_* in pJP108 and its derivative was induced with 0.05% arabinose for 1 h.

For colocalisation analysis of *M. smegmatis* ParB with DivIVA in *E. coli*, BL21(DE3) cells containing pJP108, pJP108*divIVA*_Ms_ or pCD74, and pET28a*parB_Ms_-mneon* or pACYC*parB_Ms_-mneon* (when coexpressed with pCD74) were cultured to log phase in the presence of ampicillin and chloramphenicol (in the case of pACYC). The expression of *divIVA_Cg_-mcherry* (from pCD74) and *parB_Ms_-mneon* genes was induced by the addition of 0.1 mM IPTG, while the expression of *ics-mcherry* and *mcherry-divIVA_Ms_* in pJP108 and its derivative was induced with 0.05% arabinose for 1 h.

pETDuet derivatives possessing *parB_Cc_-cfp* or *parB_Cg_-cfp* with *mcherry-ics*, *mcherry-DivIVA_Ms_*, or *divIVA_Cg_-mcherry* were cultured to log phase in the presence of ampicillin. Gene expression was induced by the addition of 0.1 mM IPTG for 1 h.

After induction, 10 μl of culture was smeared on microscopic slides and mounted with 5 μl of phosphate-buffered saline (PBS)-glycerol (1:1) solution. Cells were examined by a Leica DM6 B fluorescence microscope equipped with a 100x objective with a DFC7000 GT camera. Images were analysed using LAS X 3.6.0.20104, Fiji and “R” software (ggplot2 package; Wickham, 2009).

### ATPase Activity Assays

The ATPase activity of ParA proteins was measured using a colorimetric ATPase/GTPase activity assay kit (Sigma–Aldrich, MAK113) according to the manufacturer’s instructions. Briefly, 10 μl of 4 mM ATP was added to 30 μl reaction mixtures containing kit assay buffer and proteins of interest (diluted in assay buffer to a 2 μM concentration, in technical triplicates) in a 96-well plate. The plate was incubated for 30 min at 30°C, and then the reaction mixtures were transferred to another plate containing 200 μl of colorimetric reagent. The mixtures were further incubated for 30 min at room temperature followed by absorption measurement at 630 nm (A_630_). The obtained results were plotted against a standard curve for free phosphate. The experiment was performed in two independent replicates. All studied ParB proteins were confirmed not to exhibit ATPase activity.

### *Mycobacterium smegmatis* Growth and Microscopic Analysis

*Mycobacterium smegmatis* strains were grown either in Middlebrook liquid 7H9 medium (Difco) supplemented with 10% ADC [albumin-dextrose-catalase (BD)] and 0.05% Tween 80 or on solid 7H10 supplemented with 10% OADC [oleic acid, albumin-dextrose-catalase (BD)], 0.5% glycerol, and 0.05% Tween 80.

For growth curve analyses, *M. smegmatis* strains were inoculated from glycerol stocks and grown to log phase (OD_600_ 0.3–0.4). Next, the cultures were diluted in fresh medium with and without addition of acetamide to an OD_600_ of 0.05, and 300 μl of diluted culture was loaded into the wells of a Bioscreen C-compatible honeycomb plate. The microplate cultures were incubated at 37°C with continuous shaking using Bioscreen C [Automated Growth Curves Analysis System, Growth Curves (Alab)], and the culture optical density was measured every 20 min. The results were analysed in Excel.

For snapshot microscopy, *M. smegmatis* strains were inoculated from glycerol stocks and grown overnight. After that, cultures were diluted to OD_600_ of 0.01 in fresh medium with and without addition of acetamide and cultivated until mid-log phase (OD_600_ 0.5). For DNA staining, cells were treated with DAPI (2 μg ml^−1^) for 2 h. After centrifugation (5,000 rpm, 5 min), the cells were resuspended in PBS, and clumps were disrupted by a vortex mixer. Ten microlitres of culture was smeared on microscopic slides, dried and mounted with 5 μl of PBS-glycerol (1:1) solution. Cells were examined by a Leica DM6 B fluorescence microscope equipped with a 100x objective with a DFC7000 GT camera. Images were analysed using LAS X 3.6.0.20104 and Fiji.

### *Corynebacterium glutamicum* Strain Growth and Microscopic Analysis

For the growth analysis, *C. glutamicum* strains were grown overnight in brain heart infusion [BHI medium (OxoidTM)] with kanamycin (25 μg/ml), and the cultures were diluted with a mixture of CGXII (Keilhauer et al., 1993) and BHI medium (1:1) with kanamycin to an OD of 0.3 and cultivated overnight. Next, the overnight cultures were used to set up Bioscreen cultures with starting OD_600_—0.2 in CGXII:BHI (3:1) medium with addition of IPTG (to a final concentration of 0.1 mM) or without addition of IPTG. Three hundred microlitres of culture was loaded into the wells of a Bioscreen C-compatible honeycomb plate in three replicates for each strain. The microplate cultures were incubated at 37°C with continuous shaking using Bioscreen C [Automated Growth Curves Analysis System, Growth Curves (Alab)], and the optical density was measured every 20 min. The results were analysed in Excel.

For snapshot microscopy, *C. glutamicum* strains were grown overnight in CGXII:BHI (OxoidTM) medium with kanamycin. Next, they were cultured in 1 ml of CGXII medium to an OD_600_ of 0.5 and then induced with IPTG (final concentration of 0.1 mM) or cultured further without the addition of IPTG. Next, the cells were washed twice with PBS and resuspended in 0.2 ml of PBS with the addition of FM4-64 (10 μg/ml) and Hoechst3342 (1 μg/ml). After 15 min of incubation, the cells were mounted on microscopic slides with agarose pads and analysed using a Zeiss Axio-Imager M1 fluorescence microscope (Carl Zeiss) with an EC Plan Neofluar ×100/1.3 oil Ph3 objective and a 2.5x optovar. Images were analysed using Fiji.

### *Caulobacter crescentus* Growth and Microscopic Analysis

For microscopic experiments, *C. crescentus* strains were inoculated from glycerol stocks and grown overnight in PYE (peptone-yeast extract) medium with 1 μg/ml gentamycin. The next day, cultures were diluted 30 times to fresh medium and either induced with 0.5 mM vanillate or cultured without induction. After 5 h of further growth, 10 μl of culture was mounted on microscopic slides with agarose pads (1.2% agarose in PYE). Cells were examined by a Zeiss alpha plan achromatic 100x/1.46 oil phase 3 on an Axio Imager M2 microscope with an appropriate filter (Visitron Systems GmbH) and a cooled CCD camera (Photometrics, CoolSNAP HQ2) controlled through Metamorph (Molecular Devices).

For plating assays, *C. crescentus* strains were inoculated from glycerol stocks and grown overnight in PYE medium with 1 μg/ml gentamycin. The next day, cultures were diluted to OD = 1 in fresh PYE medium, and serial tenfold dilutions were made until 10^6^ dilution was achieved. Eight microliters of each diluted culture was transferred to PYE agar plates containing gentamycin and a specific concentration of inducer (vanillate). The plates were incubated at 30°C for 2 days.

For flow cytometry analysis, *C. crescentus* cultures were treated as previously described for microscopic experiments, but triple repetition of each strain was performed. After 5 h of cultivation, 100 μl of each culture was transferred into 900 μl of 77% ethanol on ice and stored at −20°C until further use.

To estimate chromosome number, 20 μg ml^−1^ rifampicin was added to the rest of the culture for 3 h at 30°C and then fixed with ethanol as previously described. Fixed cells were resuspended in FACS staining buffer, pH 7.2 (10 mM Tris–HCl, 1 mM EDTA, 50 mM NaCitrate, and 0.01% Triton X-100), stained with 0.5 μM SYTOX Green nucleic acid stain solution (Invitrogen), and then analysed using a BD Accuri C6 flow cytometer instrument (BD Biosciences). Flow cytometry data were acquired and analysed using CFlow Plus V1.0.264.15 software (Accuri Cytometers Inc.). A total of 20,000 cells were analysed from each biological sample. The forward scattering (FSC-A) and green fluorescence (FL1-A) parameters were used to estimate cell sizes and cell chromosome contents, respectively.

## Results

### ParA-ParB Interactions Are Conserved and Retained Between Homologues From the Same Phyla

While most bacteria employ the ParABS system to segregate their chromosomes, in some bacterial species, ParA and ParB are also involved in specific interactions with other proteins, particularly those polarly localised. Here, our aim was to understand the conservation of ParA and ParB interactions. We hypothesised that dimerisation of both ParA and ParB as well as ParA-ParB interactions are conserved and may be sustained even between proteins from distantly related species. On the other hand, we expected that the interactions with cell-pole-localised protein partners would be strictly species-specific. To explore the ParA engagement with protein partners, we chose proteins from four different microorganisms that possess the ParABS system. Two of them, *M. smegmatis* and *C. glutamicum*, are Gram-positive bacteria belonging to the Actinomycetota (formerly Actinobacteria) phylum, and two, *C. crescentus* and *R. sphaeroides*, are Gram-negative Pseudomonadota (formerly Proteobacteria).

**Figure 1:**
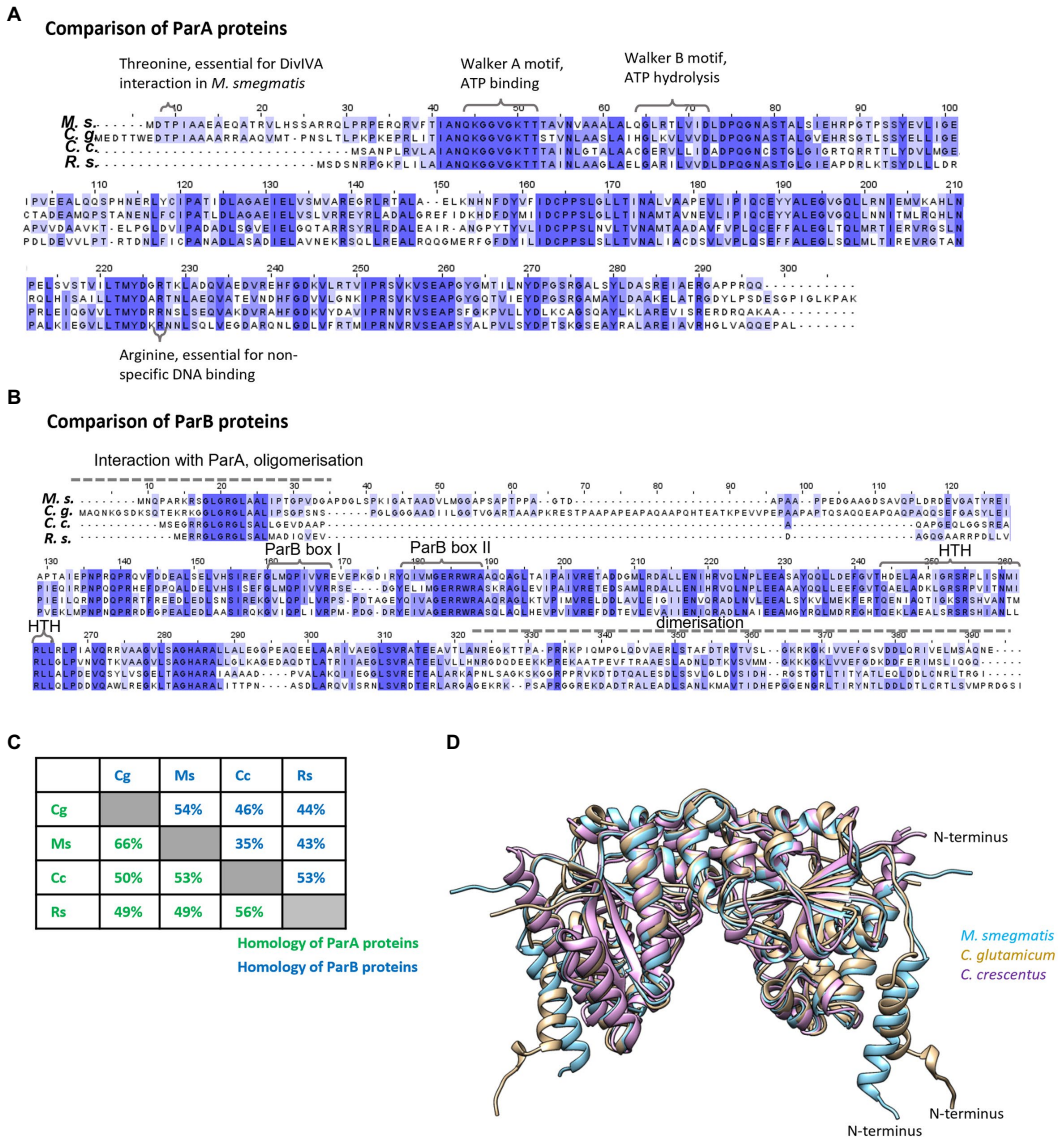
Homologies between ParA and ParB proteins from two Pseudomonadota species, *Caulobacter crescentus* and *Rhodobacter sphaeroides*, and two Actinomycetota species, *Mycobacterium smegmatis* and *Corynebacterium glutamicum.* **(A)** Sequence alignment of ParA proteins from *M. smegmatis (Ms)*, *C. glutamicum (Cg)*, and *C. crescentus (Cc)* and ParA1 from *R. sphaeroides (Rs)* aligned by Clustal Omega, visualised in Jalview, and coloured by BLOSOM62 score. Amino acids and motifs engaged in interactions are marked. **(B)** Sequence alignment of ParB proteins from *M. smegmatis (Ms)*, *C. glutamicum (Cg)*, and *C. crescentus (Cc)* and ParB1 from *R. sphaeroides* (Rs) aligned by Clustal Omega, visualised in Jalview, and coloured by BLOSOM62 score. Motifs engaged in interactions are marked. **(C)** Protein identities calculated in BLASTp. The green and blue numbers show identity between ParA and ParB proteins, respectively, from different species [*M. smegmatis* (Ms), *C. glutamicum* (Cg), *C. crescentus* (Cc), and *R. sphaeroides* (Rs)]. **(D)** The structure of ParA dimer proteins predicted by AlphaFold Colab (Jumper et al., 2021) compared and visualised by Chimera program (Pettersen et al., 2004). The blue colour indicates *M. smegmatis* ParA, brown—*C. glutamicum* ParA and violet—*C. crescentus* ParA. N-terminal of ParA from one monomer is marked.

First, we analysed the homology between the studied proteins. In both proteins ParA and ParB, the homology is highest within domains responsible for key roles in chromosome segregation (such as ATP binding, hydrolysis, DNA binding in ParA, and DNA binding in ParB; Figures 1A,B; Autret et al., 2001; Leonard et al., 2004, 2005; Hester and Lutkenhaus, 2007; Pióro et al., 2019; Jalal and Le, 2020; Kawalek et al., 2020). As expected, for both ParA and ParB homologues, proteins from species that belong to the same phylum are highly conserved (up to 66% identity and 80% similarity), while the homologues from distantly related species (e.g., *C. crescentus* and *M. smegmatis*) show lower identity (approximately 30–50%; Figure 1C). Interestingly, ParA proteins are more conserved than ParB proteins and modelling of three ParA homologues (*C. crescentus*, *C. glutamicum*, and *M. smegmatis*) shows high similarity of their spatial structures (Figure 1D). The main structural difference between ParA homologues results from 20 to 25 amino acid-long N-terminal extension in the Actinomycetota proteins. Interestingly, ParB proteins also differ substantially in the N-terminal region, which is the protein fragment engaged in interactions with ParA.

To examine the specificity of the ParA and ParB protein interactions, we employed the bacterial two-hybrid (BTH) adenylate cyclase-based system in *E. coli* (Karimova et al., 1998). The BTH system was previously used in our studies to demonstrate the interaction of *M. smegmatis* ParA with DivIVA (Ginda et al., 2013). *Escherichia coli* does not possess the ParABS system, which facilitates the use of these bacteria as heterologous hosts for studies of segregation proteins. The analysed proteins were coproduced in *E. coli* as fusions with T18 and T25 adenylate cyclase subunits. Importantly, none of the genes encoding ParB homologue contained *parS* sequence. T18 fusions with T25 (vector only) or T25 fusions with T18 (empty vector) served as negative controls. Formation of the blue colonies on indicator media (LB-X-gal plates) results from restored adenylate cyclase activity and induction of the cAMP-CRP-dependent *lac* operon and suggests the interaction between T18 and T25 fusion proteins.

**Figure 2:**
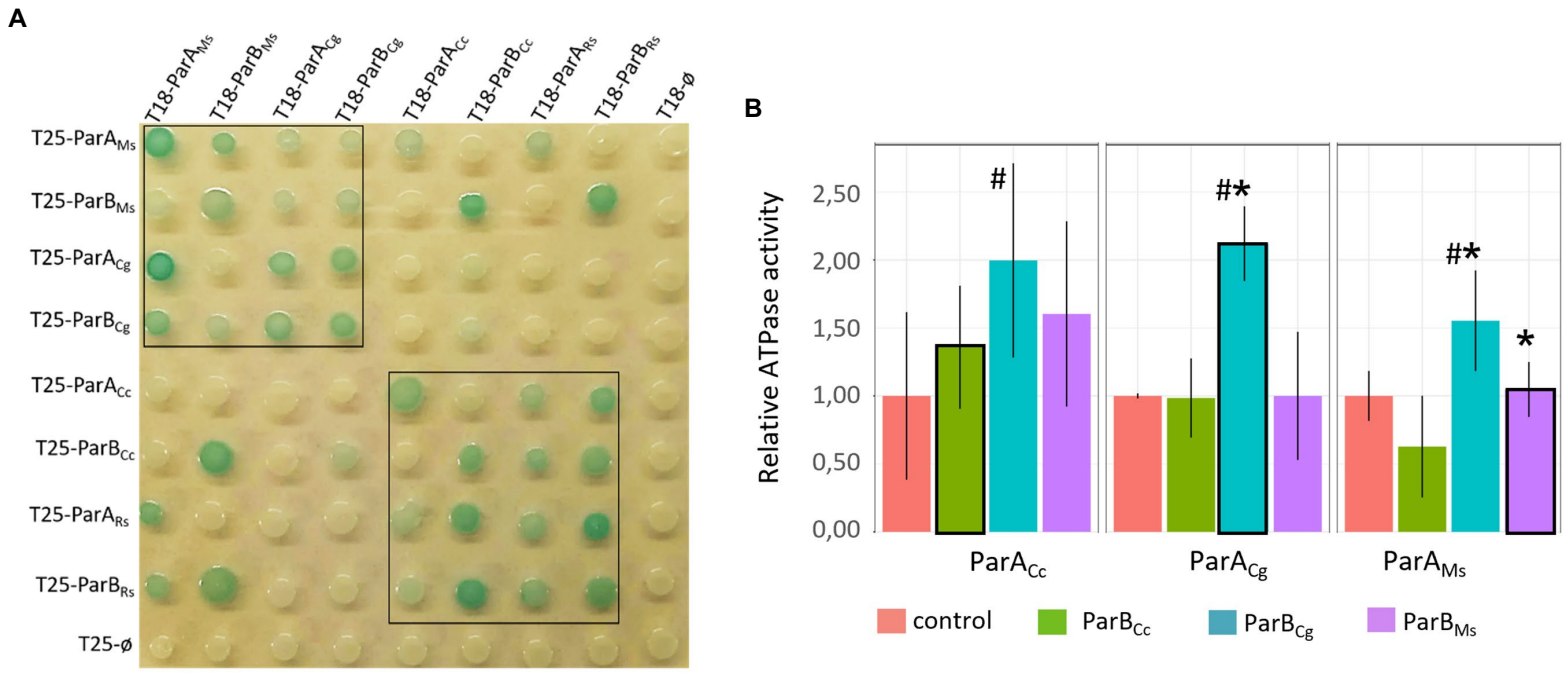
Intergenus interactions between ParA and ParB proteins.**(A)** Bacterial two-hybrid (BTH) assays testing heterodimerisation (ParA-ParA or ParB-ParB interactions) and interactions between ParA and ParB proteins from *M. smegmatis* (ParA_Ms,_ ParB_Ms_), *C. glutamicum* (ParA_Cg,_ ParB_Cg_), *C. crescentus* (ParA_Cc,_ ParB_Cc_), and ParA1 and ParB1 from *R. sphaeroides* (ParA_Rs,_ ParB_Rs_) fused to either the T18 or T25 subdomain of adenylate cyclase (as indicated) expressed from pKT25 or pUT18C plasmids. Empty pKT25 and pUT18C vectors (T25Ø or T18Ø) served as negative controls. The blue colour of the colonies indicates interactions. The black box frames the colonies co-producing proteins from the same phyla. **(B)** ATPase assay measuring the influence of different ParB proteins [*M. smegmatis* (ParB_Ms_), *C. glutamicum* (ParB_Cg_), and *C. crescentus* (ParB_Cc_)] on the ATPase activity of ParAs from *M. smegmatis* (ParA_Ms_), *C. glutamicum* (ParA_Cg_), and *C. crescentus* (ParA_Cc_). ParBs showed no ATPase activity and ATPase activities of ParA proteins in absence of ParB (shown in Supplementary Figure S1A) were normalised to 1. The plot shows the average from three replicates with SE. Stimulation of the ATPase activity of ParA by cognate ParB is marked with black frames. Statistically significant differences are marked with # (*p* < 0.15, test T-Student). Asterisks indicate ParA-ParB interactions confirmed by BTH **(A)**.

Colour of the colonies in which analysed proteins were coproduced indicated that all studied ParA and ParB proteins dimerise and they interact with their homologues from closely related species forming heterodimers (*M. smegmatis* with *C. glutamicum* proteins and *R. sphaeroides* with *C. crescentus* proteins; Figure 2A, within the black box), while interphyla interactions were less evident (pale blue colonies) or not detectable (Figure 2A). Similarly to dimerisation, the interactions between ParA and ParB were detected only for homologues from the same bacterial phyla (with one exception which is *M. smegmatis* ParA and *R. sphaeroides* ParB1 interaction). Thus, heterologous dimerisation and ParA-ParB interactions are preserved between the conserved proteins from bacteria from the same phyla.

ParA ATPase activity was earlier shown to be stimulated by ParB (Easter and Gober, 2002). Thus, we used *in vitro* assay to measure the ATPase activity of selected ParA homologues (*M. smegmatis*, closely related *C. glutamicum* and distantly related *C. crescentus*) and to examine if it is stimulated by ParB proteins from closely or distantly related species. The ATPase assay of ParA alone indicated that *M. smegmatis* ParA has the strongest activity and *C. crescentus* ParA the weakest (Supplementary Figure S1A). We also confirmed that ParB proteins had the ability to weakly enhance activity of their cognate ParAs, but the stimulation varied dependent on the ParB-ParA pair tested (Figure 2B). *Corynebacterium glutamicum* ParB was most efficient at stimulation of ParA activity as it affected its cognate ParA as well as *M. smegmatis* ParA. Surprisingly, the ATPase activity of *C. crescentus* ParA was slightly increased by all studied ParB proteins.

To summarise, we demonstrated that both ParA and ParB homologues from the same bacterial phyla form heterodimers almost as efficiently as homodimers. However, the ParA-ParB interactions are sustained for proteins from closely related species. Moreover, these interactions may be sufficient to stimulate ATPase activity of ParA.

### ParA and ParB Interactions With Polar Partner Proteins Are Genus Specific

Our earlier studies showed that the N-terminal region of *M. smegmatis* ParA is responsible for the interaction with the polar growth determinant DivIVA (Wag31; Pióro et al., 2019). Surprisingly, in closely related *C. glutamicum*, the DivIVA homologue was shown to interact with the ParB protein (Donovan et al., 2012). Interestingly, in *C. crescentus* and *R. sphaeroides*, ParB was shown to interact with MipZ (Thanbichler and Shapiro, 2006; Dubarry et al., 2019). Here, we set out to systematically analyse the interactions between ParA and ParB proteins from *M. smegmatis*, *C. glutamicum*, and *C. crescentus* as well as *R. sphaeroides* with polar partner proteins from the same and from other species using, as described above, BTH analysis (*E. coli* does not possess DivIVA and PopZ homologues).

**Figure 3:**
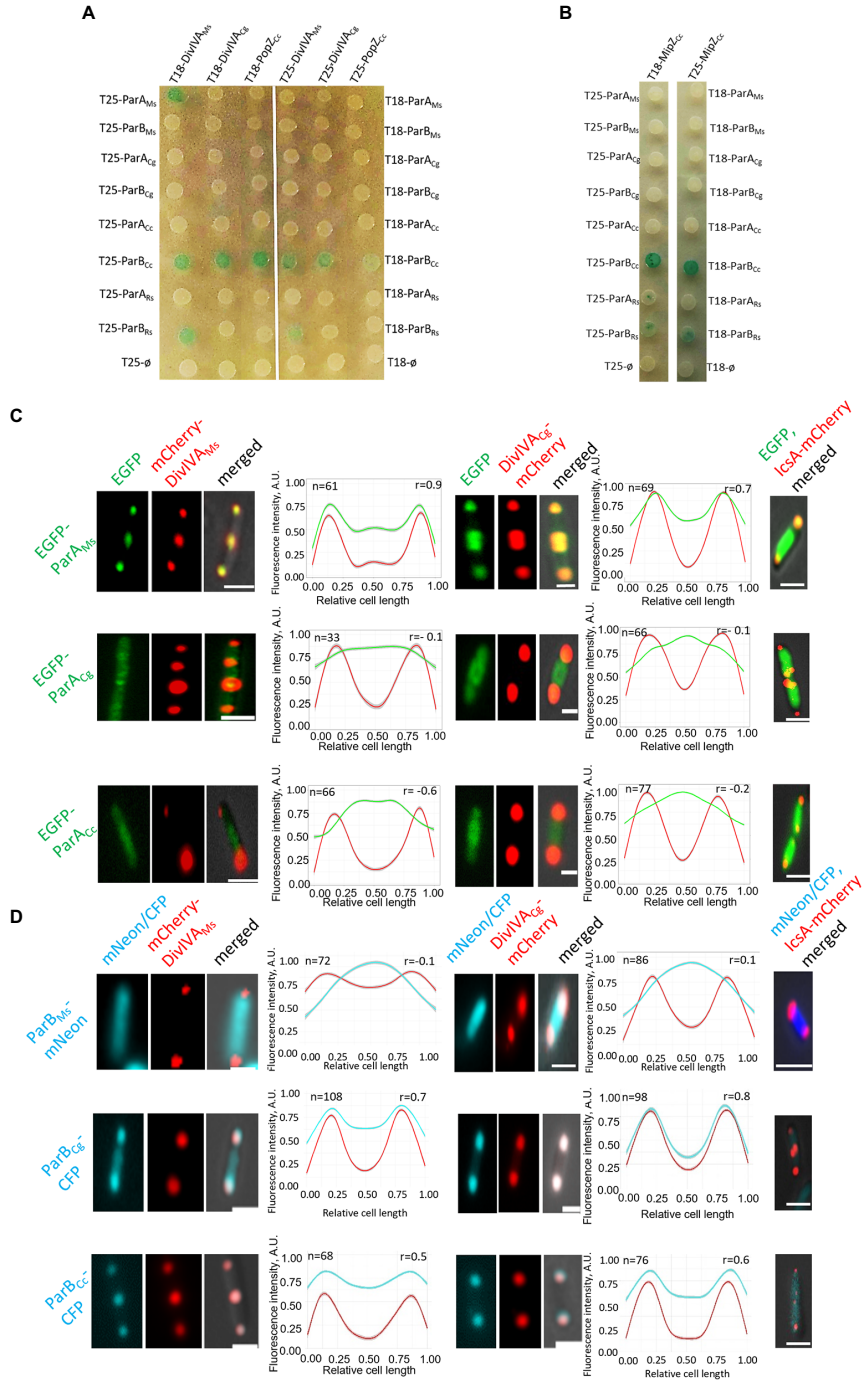
Genus-specific interactions between ParA or ParB proteins and polar partner proteins DivIVA and PopZ. **(A)** BTH assays testing interactions between *M. smegmatis* and *C. glutamicum* DivIVA (DivIVA_Ms_ and DivIVA_Cg_) or *C. crescentus* PopZ and ParA or ParB proteins from *M. smegmatis* (ParA_Ms,_ ParB_Ms_), *C. glutamicum* (ParA_Cg,_ ParB_Cg_), *C. cresentus* (ParA_Cc,_ ParB_Cc_), as well as ParA1 and ParB1 from *R. sphaeroides* (ParA_Rs,_ ParB_Rs_) fused to either the T18 or T25 subdomain of adenylate cyclase (as indicated) expressed from pKT25 or pUT18C plasmids. Empty pKT25 and pUT18C vectors (T25Ø or T18Ø) served as negative controls. The blue colour of the colonies indicates interactions. **(B)** BTH assays testing the interaction between *C. crescentus* MipZ (MipZ_Cc_) and ParA or ParB proteins from *M. smegmatis* (ParA_Ms,_ ParB_Ms_), *C. glutamicum* (ParA_Cg,_ ParB_Cg_), *C. crescentus* (ParA_Cc,_ ParB_Cc_) as well as ParA1 and ParB1 from *R. sphaeroides* (ParA_Rs,_ ParB_Rs_) fused to either the T18 or T25 subdomain of adenylate cyclase (as indicated) expressed from pKT25 or pUT18C plasmids. Empty pKT25 and pUT18C vectors (T25Ø or T18Ø) served as negative controls. The blue colour of the colonies indicates interactions. **(C)** Microscopy analysis of the colocalisation of ParA proteins from *M. smegmatis* (ParA_Ms_), *C. glutamicum* (ParA_Cg_), to *C. crescentus* (ParA_Cc_) with *M. smegmatis* and *C. glutamicum* DivIVA (DivIVA_Ms_ and DivIVA_Cg_) in *E. coli*. Representative images of *E. coli* cells coproducing EGFP-ParA (green) with mCherry-DivIVA or Ics-mCherry (red) merged with DIC (grey). *E. coli* cells coproducing Ics-mCherry (red) and one of studied fusion proteins, as indicated, served as the negative control (right panel). Scale bar: 1 μm. Graphs show green and red fluorescence intensity profiles along the cell length (number of cells analysed indicated as *n*). Lines represent models fitted using a Loess algorithm implemented in the R program. The Pearson correlation coefficient “*r*,” as the measure of colocalisation, is indicated. **(D)** Microscopic colocalisation of ParB proteins from *M. smegmatis* (ParB_Ms_), *C. glutamicum* (ParB_Cg_), and *C. crescentus* (ParB_Cc_) with *M. smegmatis* and *C. glutamicum* DivIVA (DivIVA_Ms_ and DivIVA_Cg_) in *E. coli*. Representative images of *E. coli* cells coproducing *M. smegmatis* ParB-mNeon, *C. glutamicum* and *C. crescentus* ParB-CFP and mCherry-DivIVA merged with DIC. *E. coli* cells coproducing Ics-mCherry (red) and one of studied fusion proteins, as indicated, served as the control the (right panel). Scale bar: 1 μm. Graphs show blue and red fluorescence intensity profiles along the cell length (number of cells analysed indicated as *n*). Lines represent models fitted using a Loess algorithm implemented in the R program. The Pearson correlation coefficient “*r*,” as the measure of colocalisation, is indicated.

Bacterial two-hybrid analysis revealed that *M. smegmatis* ParA interacted only with its cognate DivIVA and did not interact with *C. glutamicum* DivIVA or PopZ (Figure 3A). Markedly, the closely related *C. glutamicum* ParA did not interact with its cognate or with *M. smegmatis* DivIVA. We were also not able to detect an interaction between *C. glutamicum* ParB and its cognate DivIVA (this interaction was previously not detectable in BTH but was confirmed by another technique; Donovan et al., 2012) or *M. smegmatis* DivIVA. Surprisingly, *C. crescentus* ParB (but not ParA) interacted with all analysed polar partner proteins, i.e., both DivIVA homologues and PopZ, while *R. sphaeroides* ParB1 interacted only with *M. smegmatis* DivIVA, but not with PopZ (Figure 3A). Finally, *C. crescentus* and *R. sphaeroides* ParB, but not ParB from Actinomycetota, interacted with MipZ (Figure 3B). Moreover, BTH analysis showed that all studied polar partner proteins (both DivIVA and PopZ) form dimers or heterodimers (Supplementary Figure S1B). Thus, BTH analysis indicated that ParB homologues from *C. crescentus* and *R. sphaeroides* may interact with DivIVA but the interaction between ParA and DivIVA is specific for *M. smegmatis* proteins.

Previous studies showed that the exchange of the third amino acid in *M. smegmatis* ParA (threonine exchange for alanine) abolished its interaction with its cognate DivIVA, suggesting that the extended N-terminal fragment of this ParA homologue is involved in DivIVA binding (Pióro et al., 2019). To check whether the N-terminal fragment of *M. smegmatis* ParA was sufficient to promote DivIVA binding by the other ParA homologue, we constructed a hybrid protein that consisted of an *M. smegmatis* N-terminal ParA fragment (amino acids 1–20) fused to *R. sphaeroides* ParA and tested the interaction of the hybrid protein with *M. smegmatis* DivIVA (Supplementary Figures S1C,D). BTH system studies showed that while hybrid protein dimerized, suggesting it was functional, the N-terminal fragment of *M. smegmatis* ParA was not sufficient to induce the *R. sphaeroides* ParA interaction with *M. smegmatis* DivIVA.

Since the previously shown interactions of *C. glutamicum* ParB and DivIVA were not detectable in BTH, as a complementary approach to study the interactions between actinobacterial segregation proteins and DivIVA, we analysed their colocalisation in *E. coli* cells. For this experiment, the studied proteins were tagged with fluorescent proteins (mCherry, mNeon, CFP, or EGFP), and their localisation was studied by fluorescence microscopy. The polarly localised Ics-mCherry fusion served as the negative control (Figures 3C,D; Supplementary Figure S1E; Ptacin et al., 2010).

Further analysis confirmed the colocalisation of *M. smegmatis* ParA with *M. smegmatis* DivIVA (Ginda et al., 2013) and showed its colocalisation with *C. glutamicum* DivIVA (the latter interaction not detected in BTH; Figure 3C). Markedly, the other studied ParA homologues (*C. glutamicum* and *C. crescentus*) did not colocalise with any of the analysed DivIVA homologues, confirming that the ParA-DivIVA interaction is a specific and unique feature of *M. smegmatis* proteins.

Additionally, *E. coli* microscopy studies showed that *C. glutamicum* ParB colocalised with *C. glutamicum* DivIVA, confirming earlier observations (Figure 3D; Donovan et al., 2012). Moreover, *C. glutamicum* ParB also colocalised partially with *M. smegmatis* DivIVA, suggesting that the ability to colocalise with DivIVA is an intrinsic feature of the *C. glutamicum* ParB protein and not DivIVA. Importantly, *M. smegmatis* ParB did not colocalise with any of the studied DivIVA homologues, consistent with the BTH result. *Caulobacter crescentus* ParB colocalised with both studied DivIVA homologues, confirming the results obtained with the BTH system. The negative control experiments showed that none of the analysed ParA and ParB homologues colocalised with polarly localised Ics-mCherry (Figures 3C,D; Supplementary Figure S1E).

In summary, analyses of ParA and ParB colocalisation in *E. coli* revealed that the interactions of *M. smegmatis* ParA with DivIVA are unique to this genus. Notably, DivIVA protein is also bound by the ParB homologue from *C. crescentus* as well as *C. glutamicum* ParB.

### Overexpression of the Endogenous or Closely Related ParA Homologues Is Detrimental

Given that the interactions between ParA and ParB are retained for homologues from bacterial species belonging to the same phylum, but interactions with polar partner proteins are genus specific, we set out to investigate the importance of the latter interactions by testing the functionality of ParA protein homologues in heterologous hosts. To this end, we overproduced homologues from closely and distantly related species in wild type *C. crescentus* and *M. smegmatis* cells.

It was previously shown that overproduction of ParA in *C. crescentus* and *M. smegmatis* results in the formation of elongated cells and disturbed chromosome segregation (10% of anucleate cells; Mohl and Gober, 1997; Ginda et al., 2013). Here, we compared *C. crescentus* strains overproducing the endogenous ParA compared to those overproducing the most closely related ParA from *R. sphaeroides* and one of distantly related protein (*M. smegmatis* ParA) apart from wild type homologue (an additional gene copy expressed from a vanillate inducible promoter). Similarly, we analysed the growth of the *M. smegmatis* strain overproducing its own ParA homologue as compared to the strain overproducing closely related *C. glutamicum* or distantly related *C. crescentus* ParA (all *parA* genes were overexpressed in the wild-type background from the acetamide-induced promoter).

**Figure 4:**
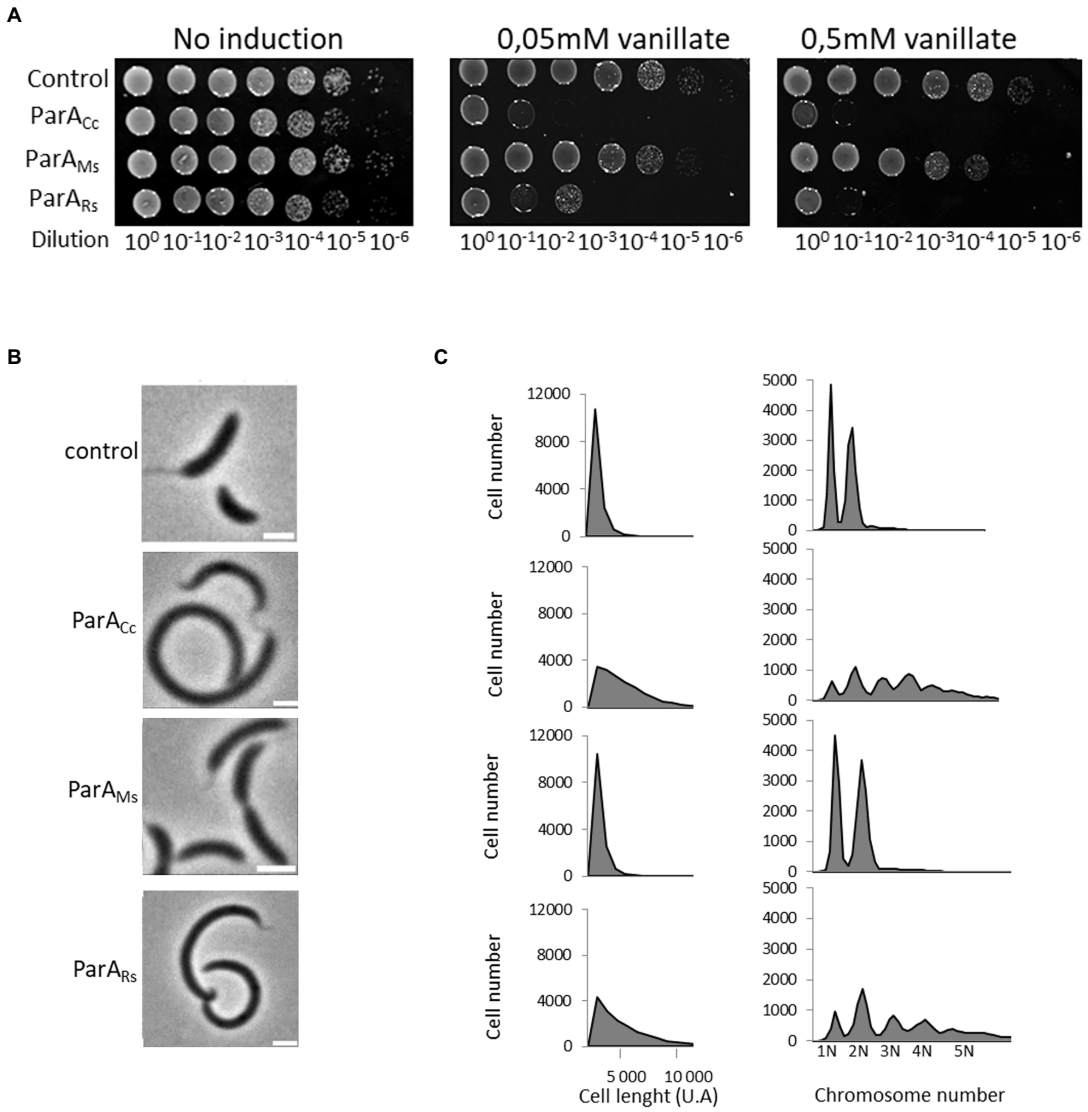
Negative effects of overproduction of the endogenous and closely related *R. sphaeroides* ParA on *C. crescentus* growth and cell morphology. **(A)** Spot plating assay to determine viability of *C. crescentus* strains overproducing *M. smegmatis* ParA (ParA_Ms_) and *R. sphaeroides* ParA1 (ParA_Rs_) compared to the *C. crescentus* strain overproducing endogenous ParA (ParA_Cc_; all in wild type background and additional *parA* copy under the control of the vanillate promoter with and without induction, as indicated). The *C. crescentus* strain containing empty vector served as a control. **(B)** Phase contrast images of *C. crescentus* strains overproducing *M. smegmatis* ParA (ParA_Ms_) and *R. sphaeroides* ParA1 (ParA_Rs_) compared to *C. crescentus* strain overproducing endogenous ParA (ParA_Cc_; as described above) and to the control strain with empty vector (control). Scale bar: 2 μm. **(C)** Flow cytometry profiles showing the cell length and chromosome numbers for *C. crescentus* strains overproducing *M. smegmatis* ParA (ParA_Ms_) and *R. sphaeroides* ParA1 (ParA_Rs_) compared to the *C. crescentus* strain overproducing endogenous (ParA_Cc_; as described above) and to the control strain with empty vector (control).

The influence of heterologous ParA proteins on *C. crescentus* growth and chromosome segregation was assayed during growth on solid medium with or without inducer and with serial dilutions of the studied strains (Figure 4A). This analysis showed that overproduction of the endogenous ParA and closely related *R. sphaeroides* homologue inhibited *C. crescentus* colony growth, while overproduction of *M. smegmatis* ParA had no effect. Microscopy analysis showed that cells of *C. crescentus* strains overproducing ParA from Alphaproteobacteria were much longer than those of the control strain (with empty vector; Figure 4B), confirming the results of growth analysis. Analysis of chromosome number by flow cytometry revealed that longer cells of strain overproducing *C. crescentus* and *R. sphaeroides* ParA contained an increased number of chromosomal copies (Figure 4C). Finally, we tested the influence of ParA overproduction on the polar localisation of MipZ-YFP in *C. crescentus* (Supplementary Figure S2). Consistent with the above-described results, overproduction of ParA from either *C. crescentus* or *R. sphaeroides*, but not *M. smegmatis*, affected the positioning of MipZ-YFP, leading to appearance of additional non-polar complexes. Thus, the overexpression of *R. sphaeroides parA* gene as additional *parA* copy had the same detrimental effect on *C. crescentus* growth as overexpression of the native gene.

**Figure 5:**
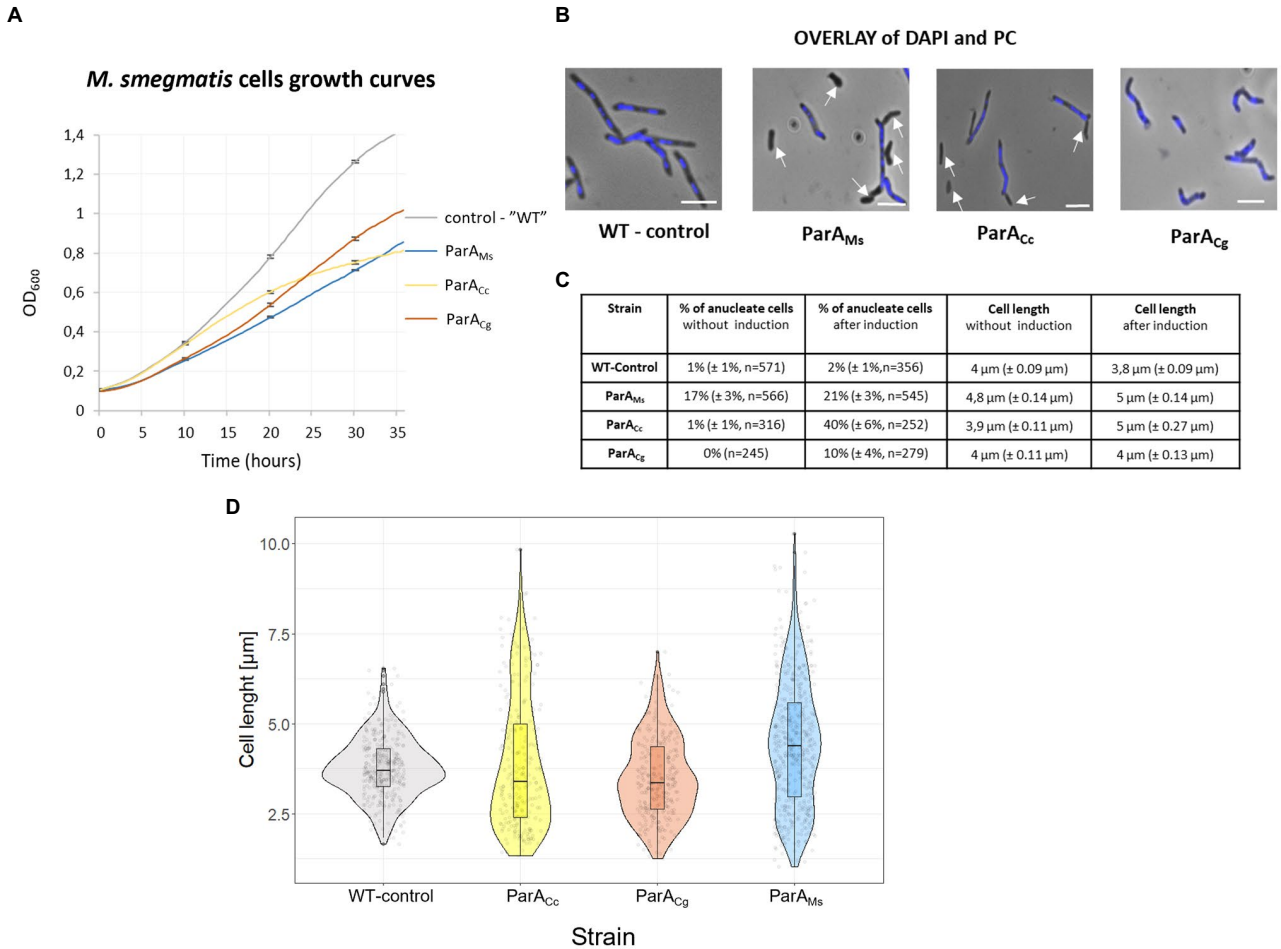
The negative effects of overproduction of the endogenous and heterologous (*C. crescentus* and *C. glutamicum*) ParA on *M. smegmatis* growth and cell morphology. **(A)** *M. smegmatis* culture growth rate of strains overproducing the endogenous (ParA_Ms_), *C. crescentus* (ParA_Cc_), and *C. glutamicum* ParA (ParA_Cg_). *parA* was overexpressed from the *parA* gene (the additional gene copy under the *p*_ami_ promoter in the pMV vector in the wild type background) induced with 1% acetamide. *M. smegmatis* WT with empty vector pMVp_ami_ served as a control. The results represent the average of three independent experiments obtained using a Bioscreen C instrument. Bars indicate SEs. **(B)** *M. smegmatis* cells overproducing endogenous (ParA_Ms_), *C. crescentus* (ParA_Cc_), or *C. glutamicum* ParA (ParA_Cg_; as descried above). Phase contrast images are merged with blue fluorescence of nucleoids stained with DAPI. WT with empty vector pMVp_ami_ served as the control. Scale bar: 3 μm. Arrows indicate anucleate cells. **(C)** Chromosome content and cell length of *M. smegmatis* cells overproducing endogenous (ParA_Ms_), *C. crescentus* (ParA_Cc_), or *C. glutamicum* ParA (ParA_Cg_; as descried above) with and without overnight induction of 1% acetamide. *M. smegmatis* WT with empty vector pMVp_ami_ served as a control. Cell length was measured only in cells possessing DNA (stained with DAPI). The experiment was performed in two independent biological replicates. The number of calculated cells is indicated as *n*. The 95% of CI was indicated as ±. **(D)** Violin plot showing the length of *M. smegmatis* cells overproducing their endogenous (ParA_Ms_), *C. crescentus* (ParA_Cc_) and *C. glutamicum* ParA (ParA_Cg_; as descried above) after overnight induction with 1% acetamide. *M. smegmatis* WT with empty vector pMVp_ami_ served as a control. The experiment was performed in two independent biological replicates. Cell length was measured in all cells (with and without nucleoids).

Overproduction of all three ParA homologues (own and heterologous proteins) in *M. smegmatis* cells decreased the liquid culture growth rate although to various extents (Figure 5A). The most efficient inhibition of culture growth was observed when the endogenous ParA protein was overproduced, while overproduction of the related *C. glutamicum* ParA resulted in lesser growth inhibition. Overproduction of *C. crescentus* ParA also affected the *M. smegmatis* growth curve, most visibly during the later growth stage. Analysis of chromosome segregation (DNA staining with DAPI) showed that overproduction of *C. glutamicum* ParA had a milder effect than overproduction of *M. smegmatis* ParA, which is consistent with the effect on the growth curve (Figures 5A–C). Surprisingly, the largest effect on chromosome segregation was observed in *M. smegmatis* overproducing *C. crescentus* ParA (40% without DNA). Overproduction of *M. smegmatis* as well as *C. crescentus* ParA homologues also increased the *M. smegmatis* cell length.

In summary, we demonstrated that *C. crescentus* growth is impaired by the overproduction of endogenous ParA as well as by closely related ParA homologue from *R. sphaeroides*. In *M. smegmatis*, the impact of overproduction was dependent on ParA homologues; the most detrimental was overproduction of endogenous protein, while overproduction of *C. glutamicum* homologue had lesser influence. This may reflect the ability of ParA homologues to engage with non-cognate ParB and indicate that some intracellular ParA interactions are maintained for closely related proteins.

### Heterologous *parA* Does Not Complement *Corynebacterium glutamicum* and *Mycobacterium smegmatis parA* Mutants

While genes encoding ParA and ParB homologues are essential in *C. crescentus*, in *C. glutamicum* and *M. smegmatis,* both genes can be deleted. A deletion mutant of *parA* in *M. smegmatis* leads to slowed growth and the formation of anucleate cells (30% of anucleate cells; Ginda et al., 2013), while abolished ParA binding to DivIVA slightly decreased the average cell length and increased the cell elongation rate (Pióro et al., 2019). As indicated by experiments above, *C. glutamicum* ParA was partially functional in *M. smegmatis* cells, however it did not interact with DivIVA, and its affinity to ParB seemed to be lower than that of cognate ParA, all of which should be expected to diminish *C. glutamicum* ParA functionality in *M. smegmatis*. We sought to confirm these results by a trying to rescue the phenotype of a *parA* deletion by complementation with *parA* from *C. glutamicum*.

**Figure 6:**
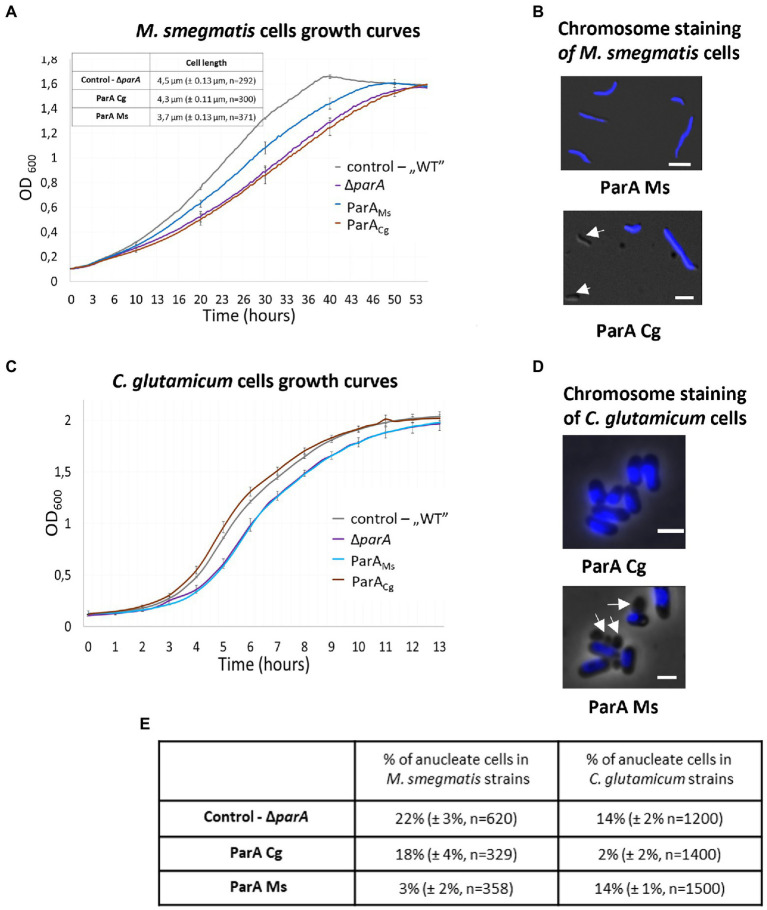
Lack of complementation of the *C. glutamicum* and *M. smegmatis parA* deletion strains with the heterologous *parA.* **(A)** Culture growth curves of the *M. smegmatis parA* deletion strain complemented with its own (ParA_Ms_) and *C. glutamicum* (ParA_Cg_) *parA* gene. *M. smegmatis* WT with empty vector pMVp_ami_ and *parA* mutant served as controls. The results represent the average of three independent experiments obtained using a Bioscreen C instrument. Bars indicate SEs. Inset: cell length of *M. smegmatis* strains obtained by microscopy. *M. smegmatis* Δ*parA* served as a control (the number of cells analysed, *n*, is indicated). **(B)** Microscopic images of *M. smegmatis parA* mutants complemented with its own (ParA_Ms_) and *C. glutamicum* (ParA_Cg_) *parA* gene. Blue fluorescence of nucleoids stained with DAPI is merged with DIC. Arrows indicate anucleate cells. Scale bar: 2 μm. **(C)** Culture growth curves of *C. glutamicum parA* mutants complemented with its own (ParA_Cg_) and *M. smegmatis* (ParA_Ms_) *parA* gene. *C. glutamicum* WT with empty vector pEKEX and *parA* mutant served as controls. The results represent the average of three independent experiments obtained using a Bioscreen C instrument. Bars indicate SEs. **(D)** Microscopy images of the *C. glutamicum parA* deletion strain complemented with its own (ParA_Cg_) and *M. smegmatis* (ParA_Ms_) *parA* gene. DIC images are merged with blue fluorescence of nucleoids stained with Hoechst3342. Arrows indicate anucleate cells. Scale bar: 2 μm. **(E)** The percentage of anucleate cells identified in the images of the DAPI-stained cells of analysed *M. smegmatis* and *C. glutamicum parA* mutant strains complemented with *M. smegmatis* (ParA_Ms_) and *C. glutamicum* (ParA_Cg_) *parA*. Deletion strains served as control. The experiment was performed in two independent biological replicates. The number of calculated cells is indicated as *n*. The 95% of CI was indicated as ±.

Analysis of the *M. smegmatis parA* deletion strain complemented with *C. glutamicum parA* showed that the production of heterologous ParA (Supplementary Figure S3) did not revert the mutant phenotype. The growth curve of *M. smegmatis* complemented with *C. glutamicum parA* was similar to that of the *parA* deletion strain (Figure 6A). Moreover, the induction of *C. glutamicum* ParA production did not restore the chromosome segregation defect compared to *parA* deletion (18% of cells were still anucleate with and without *parA* induction; Figures 6B,E). Complementation of *M. smegmatis parA* deletion with its own *parA* reduced segregation defect (3% anucleate cells after induction of *parA* gene expression). Thus, our experiment indicated that *C. glutamicum* ParA could not functionally substitute the endogenous ParA in *M. smegmatis*.

We also tested reciprocal complementation of the *C. glutamicum parA* deletion strain by *M. smegmatis parA* (Supplementary Figure S3). In *C. glutamicum*, *parA* deletion slows growth, leads to formation anucleate cells (16% in LB medium) and alters cell length (Donovan et al., 2010). Knowing that *M. smegmatis* ParA interacts with *C. glutamicum* ParB and that its ATPase activity is stimulated by this interaction, we expected that it should functionally complement *parA* deletion in *C. glutamicum*, even though unlike their native ParA it has ability to bind DivIVA.

We found that *M. smegmatis parA* did not complement *C. glutamicum parA* deletion. The growth curve of the strain with induced expression of *M. smegmatis parA* was similar to the growth curve of the *parA* deletion strain (Figure 6C). The number of anucleate cells in the *C. glutamicum parA* deletion complemented with *M. smegmatis parA* was the same as that in the *parA* deletion strain (approximately 14% with and without *parA* induction). In contrast, induced expression of *C. glutamicum parA* reverted *parA* deletion phenotype (number of anucleate cells lowered to 2%; Figures 6D,E). Interestingly, although the number of anucleate cells in the *C. glutamicum parA* deletion strain complemented with *M. smegmatis parA* was the same as that in the *parA* deletion, the fractions of cells with 1 and 2 ParB-YPet complexes differed significantly, with more cells with 1 or 2 ParB complexes (34 and 44%, respectively) observed in the complemented strain than in the *parA* deletion strain (17 and 35%; Supplementary Figure S4). This may indicate that the production of *M. smegmatis* ParA in *C. glutamicum* may impair the overlapping chromosome replication cycles characteristic of *C. glutamicum* cells.

In summary, the complementation analyses show that even highly homologous ParA proteins, whose heterologous interactions are at least partially sustained, are not functional enough to convey chromosome segregation. This observation highlights the significance of all the species-specific interactions of chromosome segregation proteins.

## Discussion

The bacterial chromosome segregation proteins ParA and ParB from various bacteria, although share basic biochemical properties, exhibit the ability to engage in unique, species-specific interactions. Our aim was to explore how conserved the interactions are between ParA and ParB and to determine whether they are sufficient to sustain the *in vivo* functionality of heterologous proteins. It should be kept in mind that using *E. coli* systems (BTH, co-localisation studies) for interaction studies may enhance or diminish the intermolecular interactions due to the lack of the biological context. However, application of two complementary systems allowed us to study combinations of interactions and delivered consistent results. Moreover, the application of the native strains as the host in overexpression or complementation analysis delivered information on the exogenous protein functionality (Table 1).

**Table 1:** Summary of ParA and ParB proteins functionality in native and heterologous environment.

ParA/ParB functionality	Native	Distantly related (between phyla)	Closely related (within the phylum)
Dimerisation	Confirmed for all ParA and ParB proteins	Only: ParB_Ms_—ParB_Cc_ ParB_Ms_—ParB_Rs_	ParA_Rs_-ParA_Cc_ ParA_Cg_-ParA_Ms_ ParB_Rs_-ParB_Cc_ ParB_Cg_-ParB_Ms_
ParA interaction with ParB	Confirmed for all species	Not detected	ParA_Rs_-ParB_Cc_ ParB_Rs_-ParA_Cc_ ParA_Cg_-ParB_Ms_ ParB_Cg_-ParA_Ms_
Interaction with polar proteins	ParA_Ms_-DivIVA_Ms_ ParB_Cg_-DivIVA_Cg_ ParB_Cc_-PopZ_Cc_	ParB_Cc_-DivIVA_Ms_ ParB_Cc_-DivIVA_Cg_ ParB_Rs_-DivIVA_Ms_	ParA_Ms_-DivIVA_Cg_ ParB_Cg_-DivIVA_Ms_
Effects of ParA overproduction	Endogenous ParA—growth inhibition and segregation defect	ParA_Cc_ in *Mycobacterium smegmatis* background—growth inhibition and segregation defect at prelonged induction ParA_Ms_ in *C. crescentus*—no effect	ParA_Rs_ in *C. crescentus* background ParA_Cg_ in *M. smegmtis* background Growth inhibition and chromosome segregation defects
Complementation of *parA* deletion strain by *parA*	Fully complemented	Not tested	Lack of complementation

We showed that ParA and ParB proteins from different bacterial species form heterodimers (or higher order oligomers), particularly protein pairs from the same bacterial phylum. The ParA-ParB interactions are predominantly maintained within pairs of proteins from the same bacterial phyla. While these observations are consistent with the expectations, they are not fully supported by the observed influence of ParB on the ATPase activity of ParA. The difference in the N-terminal fragment of ParB, which was shown to be engaged in interaction with ParA (Autret et al., 2001), may account for the observed differences in ParB-ParA interactions. ParB ability to interact with ParA is supposed to affect formation of ParA gradient and efficiency of chromosome segregation (Vecchiarelli et al., 2014). Our analysis suggests the species-specific tuning of ParB—ParA interactions. Recently similar significance of species dependent interactions between ParB and SMC was reported (Bock et al., 2021).

An interesting feature of the ParAB system is its species-specific adjustment to interactions with polarly localised proteins. While in *M. smegmatis* ParA interacts with polar DivIVA, the closely related *C. glutamicum* ParA does not share this ability. In contrast, as shown before (Donovan et al., 2012) in *C. glutamicum*, ParB is involved in the interaction with DivIVA. We also detected ability of *C. crescentus* and *R. sphaeroides* ParB to interact with DivIVA. This interaction may result from similar structural features of DivIVA and PopZ, namely their ability to form higher order branched filamentous superstructure (Bowman et al., 2013; Holmes et al., 2016). We expected that the N-terminal extension of *M. smegmatis* ParA may play a role in species-specific interactions with DivIVA. Notably, 20 N-terminal amino acids in *M. smegmatis* ParA are not present in ParA homologues from other phyla and are only partially similar to the elongated N-terminus of *C. glutamicum* ParA. Previously, we showed that the exchange of the third amino acid in ParA abolishes its binding to DivIVA (Pióro et al., 2019). However, simply fusing 20 N-terminal amino acids from *M. smegmatis* ParA to *R. sphaeroides* protein did not promote its interactions with *M. smegmatis* DivIVA, indicating that the interaction interface is more complex and extends beyond the N-terminal fragment of *M. smegmatis* ParA. Markedly, *M. smegmatis* ParA is recruited to both *M. smegmatis* and *C. glutamicum* DivIVA. However, *M. smegmatis* DivIVA also weakly interacted with *C. glutamicum* ParB, which efficiently bound its cognate DivIVA. This indicates that the ability to bind DivIVA seems to be an intrinsic feature of the ParA or ParB protein.

**Figure 7:**
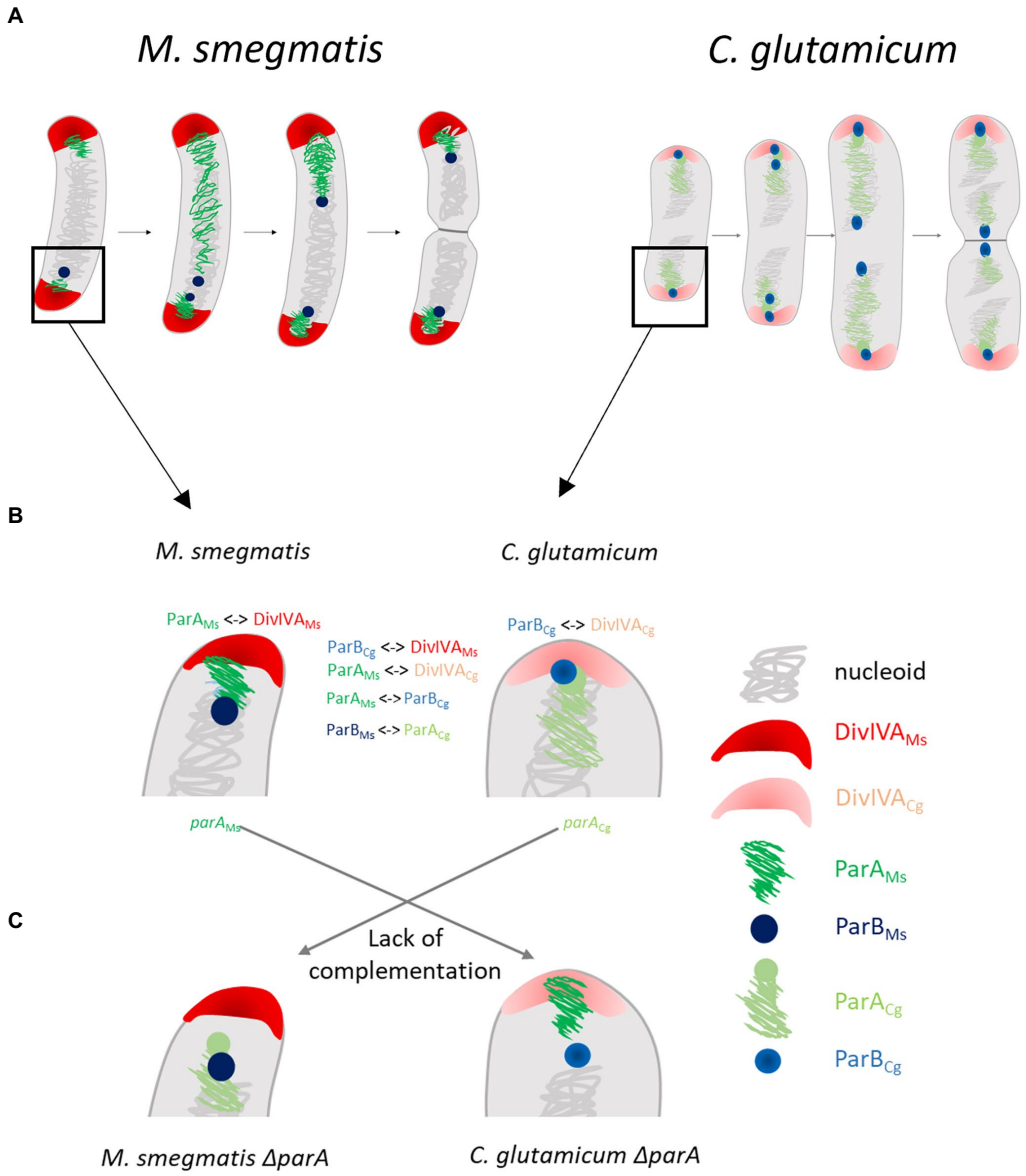
The species specific interactions of segregation and polarly localised proteins in Actinobacteria are critical for efficient chromosome segregation and may be linked with the different *oriC* positioning. **(A)** Scheme of differences in *oriC* positioning in closely related *Mycobacterium smegmatis* and *Corynebacterium glutamicum* cells which undergo non-overlapping (1 or 2 *oriCs* in cell in some distance from the pole) or overlapping rounds of replication (2 or 4 *oriCs*, polarly localised), respectively. (**B)** The interactions of segregation proteins and polar partner proteins (marked with symbol <−>) and detailed *oriC* positioning (polar or in distance to the pole). **(C)** The lack of complementation of *parA* deletion phenotype with heterologous *parA*.

The nonpreserved interactions between ParA and ParB from different bacterial phyla presumably account for the lack of functionality of distantly related homologues in heterologous systems (Figure 7). While the effects of overproduction of the highly homologous protein are similar to the effects of overproduction of cognate protein, although they may be less enhanced, the overproduction of distantly related proteins, i.e., *M. smegmatis* in *C. crescentus*, had no effect on growth and chromosome segregation. An exception to this rule was the toxic effect observed upon overproduction of *C. crescentus* ParA in the *M. smegmatis* background.

Markedly, *C. glutamicum* ParA was not able to functionally substitute for the *M. smegmatis* homologue and in any extent did not restore the *parA* deletion phenotype. This finding could be considered surprising since *C. glutamicum* ParA is highly homologous to *M. smegmatis* protein, it interacted with *M. smegmatis* ParB in BTH system and its overproduction influenced *M. smegmatis* growth, similarly as overproduction of native protein even though to a lesser extent. Moreover, the lack of interaction between *C. glutamicum* ParA and *M. smegmatis* DivIVA cannot account for the severe segregation defect of the complemented strain since disruption of the DivIVA-ParA interaction was shown to only modestly affect chromosome segregation (Pióro et al., 2019). Speculatively, the lack of complementation could be explained by the inefficient enhancement of *C. glutamicum* ParA ATPase activity by *M. smegmatis* ParB.

However, even more surprisingly, the *C. glutamicum parA* deletion strain could not be complemented by homologous *M. smegmatis* ParA. The *M. smegmatis* ParA could be expected to be able to functionally substitute *C. glutamicum* ParA since its ATPase activity was somewhat enhanced by *C. glutamicum* ParB. However, *M. smegmatis* ParA, unlike *C. glutamicum* ParA, was shown to interact with *C. glutamicum* DivIVA, which might disturb the protein interaction network, specifically ParB-DivIVA interactions. This may suggest that all the complex interactions of segregation proteins, including those with polar protein partners, are crucial for their full functionality *in vivo*.

The surprising difference in DivIVA interaction partners (ParA in *M. smegmatis* and ParB in *C. glutamicum*) may explain not only the lack of heterologous protein functionality but also the different *oriC* region positions in these closely related species. It is worth mentioning that genus-specific interactions of ParA with TipN and PopZ in *C. crescentus* or bactofilins in *Myxoccocus xanthus* are crucial for specific *oriC* positioning (Ptacin et al., 2010; Schofield et al., 2010; Lin et al., 2017). Thus, in fast-growing *C. glutamicum* with overlapping replication cycles, ParB anchors *oriC* close to the cell pole (Donovan et al., 2012; Böhm et al., 2017), while in relatively slow-growing *M. smegmatis,* ParA recruitment to DivIVA positions *oriC* at some distance from the pole (Figure 7; Ginda et al., 2013; Pióro et al., 2019). Speculatively, ParA or, alternatively, ParB interaction with DivIVA may be one of the cell cycle checkpoints. This notion may be supported by the change in the number of ParB complexes in *C. glutamicum* producing *M. smegmatis* ParA instead of their own homologue. In those cells, ParA would likely be recruited to DivIVA, possibly affecting the DivIVA-ParB interaction and speculatively modifying the cell cycle. Thus, we infer that ParA or ParB interactions with DivIVA may be related to the requirements of the cell cycle or may even manifest some cell cycle features.

Our studies constitute the first interspecies analysis of segregation proteins and polarly localised protein interactions. We show the significance of the fine-tuning of the ParB-ParA interaction, which may be related to the stimulation of ParA ATPase activity. We also demonstrate that genus-specific recruitment of ParA or ParB to DivIVA is critical for efficient chromosome segregation and growth. Our studies reveal the interaction network of segregation proteins that is critical to their function and demonstrate that even closely related ParA and ParB homologues are engaged in genus-specific interactions related to specific requirements of the particular bacterial species cell cycle.

## Data Availability Statement

The original contributions presented in the study are included in the article/Supplementary Material, further inquiries can be directed to the corresponding authors.

## Author Contributions

MP, MBr, and DJ contributed to conception and design of the study. MP wrote original draft, performed analysis, and visualized data. MP, IM, TŁ, AG, and PJ performed experiments. KB, MBr, JA, PV, and MBe delivered resources. MP, IM, and DJ supervised students. PV, MBr, and DJ reviewed and edited manuscript. DJ administrated project and raised funds. All authors contributed to the article and approved the submitted version.

## Funding

This work was funded by OPUS grant 2017/27/B/NZ1/00823 and partially by Harmonia grant 2014/14/M/NZ1/00076 (results presented in Figure 5; Supplementary Figure S2) from the National Science Centre, Poland. DJ visit to the Marc Bramkamp laboratory at Ludwig Maximilian University, Munich, was funded by the Polish National Agency for Academic Exchange (NAWA, project number PPN/BEK/2018/1/00399). MB acknowledges funding from Deutsche Forschungsgemeinschaft (DFG) grant BR2915/6-2. The cost of publication was financed by University of Wrocław.

## Conflict of Interest

The authors declare that the research was conducted in the absence of any commercial or financial relationships that could be construed as a potential conflict of interest.

## Publisher’s Note

All claims expressed in this article are solely those of the authors and do not necessarily represent those of their affiliated organizations, or those of the publisher, the editors and the reviewers. Any product that may be evaluated in this article, or claim that may be made by its manufacturer, is not guaranteed or endorsed by the publisher.

## Supplementary Material

Supplementary Figure S1 ParA protein interactions. **(A)** ATPase activity of sole ParA proteins measured in μmoles of free phosphate/min/L (U/L) according to the manufacturer’s protocol (Sigma-Aldrich), indicating the greatest activity of the *Mycobacterium smegmatis* ParA protein and the lowest activity in the case of the *C. crescentus* ParA protein. **(B)** BTH assays testing interactions between *M. smegmatis* (DivIVA_Ms_) and *Corynebacterium glutamicum* (DivIVA_Cg_) DivIVA as well as *C. crescentus* (PopZ_Cc_) PopZ proteins fused to either the T18 or T25 subdomain of adenylate cyclase (Cya, as indicated) expressed from pKT25 or pUT18C plasmids. Empty pKT25 and pUT18C vectors (T25Ø or T18Ø) served as negative controls. The blue colour of the colonies indicates interactions. **(C)** Amino acid sequences of the N-terminus regions of the *M. smegmatis* and *R. sphaeroides* ParA proteins. The hybrid protein consists of the first 20 amino acids of *M. smegmatis* ParA (marked in blue), and the remaining protein is the *R. sphaeroides* sequence. **(D)** BTH assays testing the interaction of hydrid ParA fused with the T25 subdomain of adenylate cyclase with *M. smegmatis* DivIVA (DivIVA_Ms_) or ParA (ParA_Ms_) fused with T18. The positive control is *M. smegmatis* T25-ParA with T18-DivIVA. The blue colour of the colonies indicates interactions. **(E)** Lack of colocalisation of *M. smegmatis* EGFP-ParA and ParB-mNeon (ParA_Ms,_ ParB_Ms_), *C. glutamicum* EGFP-ParA, ParB-CFP (ParA_Cg_, ParB_Cg_), and *C. crescentus* EGFP-ParA, ParB-CFP (ParA_Cc_, ParB_Cc_) with IcsA-mCherry. Graphs show green/blue and red fluorescence intensity profiles along the cell length (number of cells analysed indicated as n). Lines represent models fitted using a Loess algorithm implemented in the R program. The Pearson correlation coefficient “r,” as the measurement of colocalisation, is indicated.

Supplementary Figure S2 Localisation of MipZ-YFP in *C. crescentus* cells. *C. crescentus* cells producing MipZ-YFP and overexpressing *C. crescentus* (ParA_Cc_), *Mycobacterium smegmatis* (ParA_Ms_) *parA* genes or *R. sphaeroides parA1* (ParA_Rs_) with and without induction with vannilate. Florescence images of MipZ-YPF fluorescence (yellow) merged with DIC. *C. crescentus* strain MT97 served as a control. Scale bar 2 μm.

Supplementary Figure S3 Confirmation of ParA proteins production in *Mycobacterium smegmatis* and *Corynebacterium glutamicum* strain. **(A)** Western blot analysis of *M. smegmatis* Δ*parA* strains overexpressing own (Ms) and heterologous *parA* from *C. glutamicum* (Cg). Δ*parA* strain and mc2 served as controls. Strains were cultured overnight with 10% acetamide induction (marked as +) or without (−). Cells were lysed by sonication and 100 μg of total proteins were subjected to 10% SDS-PAGE electrophoresis and then transferred on nitrocellulose blotting membrane. The membrane was incubated with rabbit serum containing anti ParA_Ms_ antibody. As secondary antibody anti-rabbit IgG conjugated with alkaline phosphatase was used. Prestained protein ladder (Thermo Scientific) was used as a marker (marked as M). **(B)** Western blot analysis of cell lysate from *C. glutamicum* Δ*parA* strains complemented with own (ParACg) or heterologous *parA* from *M. smegmatis* (ParAMs), two clones of each strain were analysed. *C. glutamicum* Δ*parA* strain was used as the control (Δ*parA*). Cultures at logarithmic phase were induced with 0.05 mM IPTG for 2 h. Cells were lysed by sonication and 100 μg of proteins were subjected to 10% SDS-PAGE electrophoresis and then transferred on nitrocellulose blotting membrane. The membrane was incubated with rabbit serum containing anti-ParA_Ms_ antibody. As secondary antibody anti-rabbit IgG conjugated with alkaline phosphatase was used. Prestained protein ladder Thermo Scientific was used as a marker (marked as M).

Supplementary Figure S4 ParB complexes in *Corynebacterium glutamicum parA* mutant strains. C. *glutamicum parA* deletion strain complemented with native *parA* gene (ParA_Cg_) and *Mycobacterium smegmatis parA* (ParA_Ms_). Upper panel: microscopy images of examples of *C. glutamicum* cells producing ParB-YPet (yellow) with membranes stained with FM4-64 (red). Lower panel: percentage of cells possessing a specific number of ParB complexes. Scale bar: 1 μm.
