# Supplementary material for: Genus-Specific Interactions of Bacterial Chromosome Segregation Machinery Are Critical for Their Function
Source: Front Microbiol. 2022 Jul 6;13:928139. doi: 10.3389/fmicb.2022.928139 (PMC9298525; doi:10.3389/fmicb.2022.928139)
Supplement: Supplementary Figure S1 — ParA protein interactions. (A) ATPase activity of sole ParA proteins measured in μmoles of free phosphate/min/L (U/L) according to the manufacturer’s protocol (Sigma-Aldrich), indicating the greatest activity of the Mycobacterium smegmatis ParA protein and the lowest activity in the case of the C. crescentus ParA protein. (B) BTH assays testing interactions between M. smegmatis (DivIVAMs) and Corynebacterium glutamicum (DivIVACg) DivIVA as well as C. crescentus (PopZCc) PopZ proteins fused to either the T18 or T25 subdomain of adenylate cyclase (Cya, as indicated) expressed from pKT25 or pUT18C plasmids. Empty pKT25 and pUT18C vectors (T25Ø or T18Ø) served as negative controls. The blue colour of the colonies indicates interactions. (C) Amino acid sequences of the N-terminus regions of the M. smegmatis and R. sphaeroides ParA proteins. The hybrid protein consists of the first 20 amino acids of M. smegmatis ParA (marked in blue), and the remaining protein is the R. sphaeroides sequence. (D) BTH assays testing the interaction of hydrid ParA fused with the T25 subdomain of adenylate cyclase with M. smegmatis DivIVA (DivIVAMs) or ParA (ParAMs) fused with T18. The positive control is M. smegmatis T25-ParA with T18-DivIVA. The blue colour of the colonies indicates interactions. (E) Lack of colocalisation of M. smegmatis EGFP-ParA and ParB-mNeon (ParAMs, ParBMs), C. glutamicum EGFP-ParA, ParB-CFP (ParACg, ParBCg), and C. crescentus EGFP-ParA, ParB-CFP (ParACc, ParBCc) with IcsA-mCherry. Graphs show green/blue and red fluorescence intensity profiles along the cell length (number of cells analysed indicated as n). Lines represent models fitted using a Loess algorithm implemented in the R program. The Pearson correlation coefficient “r,” as the measurement of colocalisation, is indicated. [file Data_Sheet_1.PDF]

Figure S1

A.

ATPase activity assay	Enzyme activity (µmol of free phosphate/minute/L)
ParA Ms	3,77
ParA Cc	1,7
ParA Cg	2,27

B.

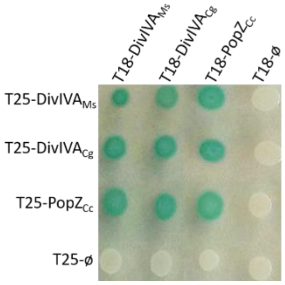

C.

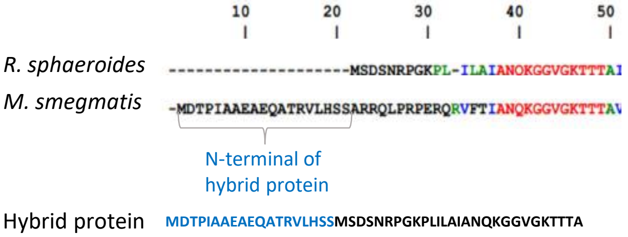

D.

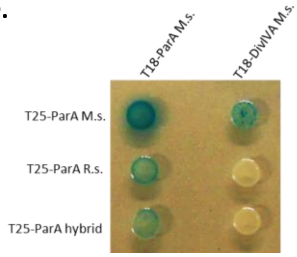

E.

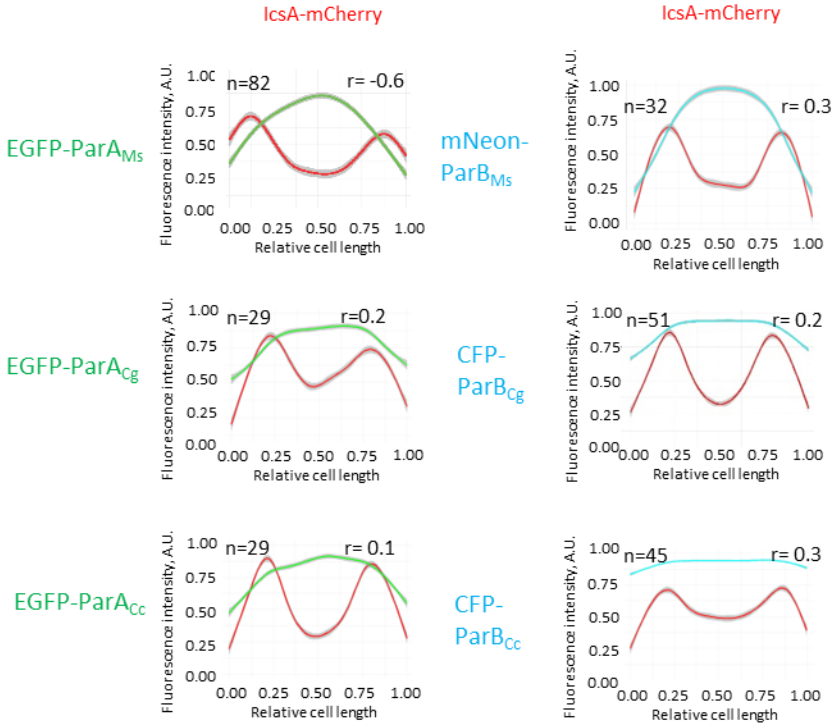

Figure S2

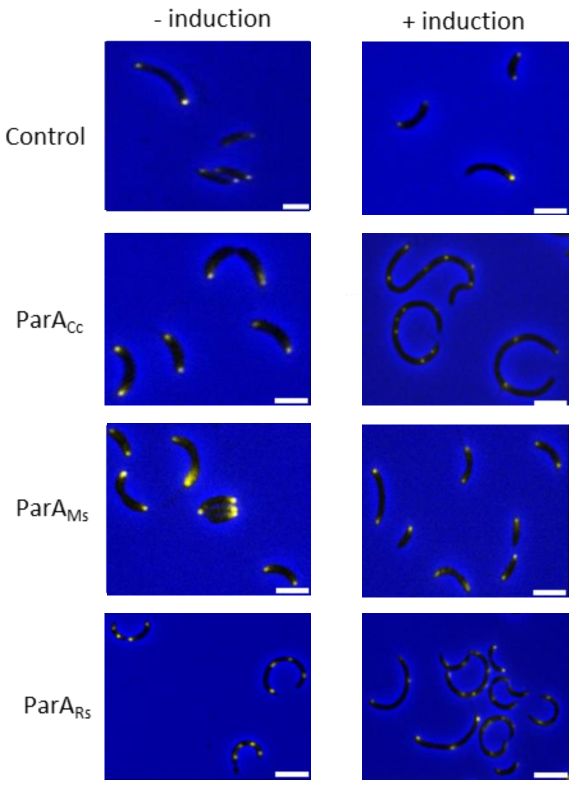

Figure S3

A. Confirmation of expression of *parA*$_{Ms}$ and *parA*$_{Cg}$ in *M. smegmatis* Δ*parA background*

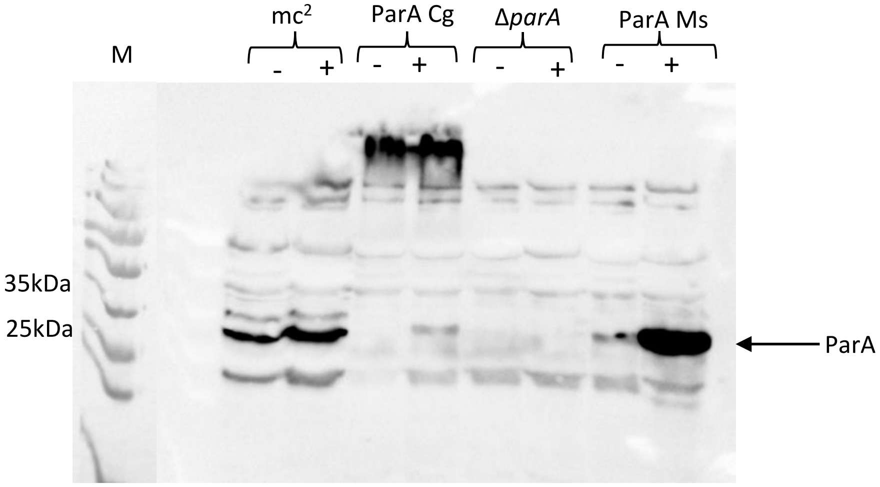

B. Confirmation of expression of *parA*$_{Ms}$ and *parA*$_{Cg}$ in *C. glutamicum* Δ*parA background*

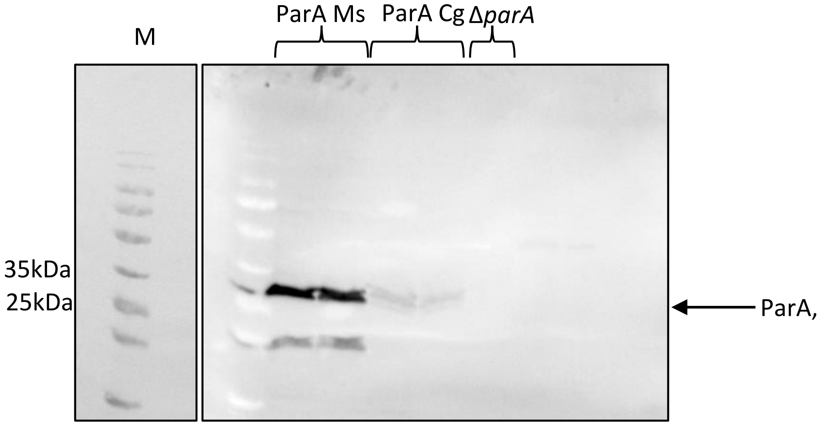

Figure S4

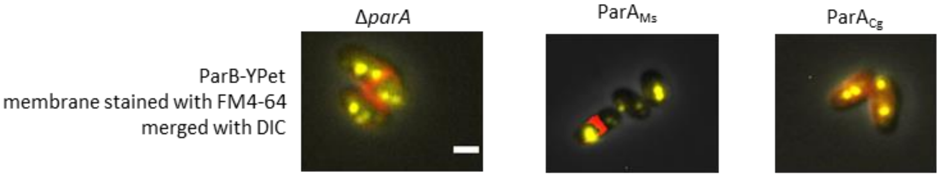

C. glutamicum strain	1 ParB complex (%)	2 ParB complexes (%)	3 and more ParB complexes (%)
ΔparA	17%	35%	48%
$parA_{Ms}$	34%	44%	22%
$parA_{Cg}$	19%	42%	39%
