# Supplementary material for: Genus-Specific Interactions of Bacterial Chromosome Segregation Machinery Are Critical for Their Function
Source: Front Microbiol. 2022 Jul 6;13:928139. doi: 10.3389/fmicb.2022.928139 (PMC9298525; doi:10.3389/fmicb.2022.928139)
Supplement: Supplementary file 2 [file Data_Sheet_2.docx]

**Table S1. Constructs and strains used in this study**

| **Constructs**  **and strains** | **Relevant genotype** | **Source** |
| --- | --- | --- |
| **Constructs used in BTH system in *E. coli*** | | |
| pUT18Cø | pUC replicon, p_lac_ promoter, ampicillin resistance, IPTG-inducible, T18 fragment of *cyaA* gene (encoding 225 to 399 amino acids) | (Karimova et al., 1998) |
| pKT25ø | P15A replicon, p_lac_ promoter, kanamycin resistance, IPTG-inducible T25 fragment of *cyaA* gene (encoding first 224 amino acids) | (Karimova et al., 1998) |
| pKT25*parA*_Ms_ | pKT25 containing *M. smegmatis parA* gene | (Ginda et al., 2013) |
| pUT18C*parA*_Ms_ | pUT18C containing *M. smegmatis parA* gene | (Ginda et al., 2013) |
| pKT25*parB*_Ms_ | pKT25 containing *M. smegmatis parB* gene | (Ginda et al., 2013) |
| pUT18C*parB*_Ms_ | pUT18C containing *M. smegmatis parB* gene | (Ginda et al., 2013) |
| pKT25*divIVA*_Ms_ | pKT25 containing *M. smegmatis divIVA* gene | (Ginda et al., 2013) |
| pUT18C*divIVA*_Ms_ | pUT18C containing *M. smegmatis divIVA* gene | (Ginda et al., 2013) |
| pKT25*parA*_Cg_ | pKT25 containing *C. glutamicum parA* gene | This study |
| pUT18C*parA*_Cg_ | pUT18C containing *C. glutamicum parA* gene | This study |
| pKT25*parB*_Cg_ | pKT25 containing *C. glutamicum parB* gene | (Böhm et al., 2020) |
| pUT18C*parB*_Cg_ | pUT18C containing *C. glutamicum parB* gene | Böhm et al., 2020) |
| pKT25*divIVA*_Cg_ | pKT25 containing *C. glutamicum divIVA* gene | Böhm et al., 2020) |
| pUT18C*divIVA*_Cg_ | pUT18C containing *C. glutamicum divIVA* gene | Böhm et al., 2020) |
| pKT25*parA*_Cc_ | pKT25 containing *C. crescentus parA* gene | This study |
| pUT18C*parA*_Cc_ | pUT18C containing *C. crescentus parA* gene | This study |
| pKT25*parB*_Cc_ | pKT25 containing *C. crescentus parB* gene | This study |
| pUT18C*parB*_Cc_ | pUT18C containing *C. crescentus parB* gene | This study |
| pKT25*popZ*_Cc_ | pKT25 containing *C. crescentus popZ* gene | This study |
| pUT18C*popZ*_Cc_ | pUT18C containing *C. crescentus popZ* gene | This study |
| pKT25*parA*_Rs_ | pKT25 containing *R. sphaeroides parA* gene | J. Armitage lab stock, University of Oxford, Oxford |
| pUT18C*parA*_Rs_ | pUT18C containing *R. sphaeroides parA* gene | J. Armitage lab stock, University of Oxford, Oxford |
| pKT25*parB*_Rs_ | pKT25 containing *R. sphaeroides parB* gene | J. Armitage lab stock, University of Oxford, Oxford |
| pUT18C*parB*_Rs_ | pUT18C containing *R. sphaeroides parB* gene | J. Armitage lab stock, University of Oxford, Oxford |
| pUT18C*mipZ* | pUT18C containing *C. crescentus* *mipZ* gene | This study |
| pKT25*mipZ* | pKT25 containing *C. crescentus* *mipZ* gene | This study |
| pKT25*hybrid_parA* | pKT25 containing *R. sphaeroides* and *M. smegmatis parA* hybrid gene | This study |
| **Constructs used for protein overproduction in *E. coli*** | | |
| pET21*parB_Ms_* | pET21a ampicillin resistance, His-tag vector containing *M. smegmatis parB* gene | K. Ginda, unpublished, D. Jakimowicz lab stock, University of Wroclaw |
| pGEX6P2*parA_Ms_* | pGEX6P2 ampicillin resistance, GST-tag vector containing *M. smegmatis parA* gene | (Jakimowicz et al., 2007) |
| pGEX6P2*parB_Cc_* | pGEX6P2 ampicillin resistance, GST-tag vector containing *C. crescentus parB* gene | This study |
| pET21a*parA_Cc_* | pET21a ampicilin resistance, vector containing  *C. crescentus parA* gene | Gift from M. Thanbichler, Max Planck, Marburg (Corrales-Guerrero et al., 2020) |
| pET28*parA_Cc_* | pET28a kanamycin resistance, His-tag vector containing *C. crescentus parA* gene | This study |
| pET16b*parB_Cg_* | pET16b ampicillin resistance, His-tag vector containing *C. glutamicum parB* gene | (Donovan et al., 2010) |
| pET16b*parA_Cg_* | pET16b ampicillin resistance, His-tag vector containing *C. glutamicum parA* gene | M. Bramkamp lab stock, Kiel University, unpublished |
| **Constructs used for** **colocalisation microscopy in *E. coli*** | | |
| pCD02 | pETDuet, ampicillin resistance vector, containing *cfp* gene | (Sieger et al., 2013) |
| pCD74 | pETDuet containing *C. glutamicum* *divIVA* gene fused with *mcherry* | (Sieger & Bramkamp, 2015) |
| pCD17 | pETDuet containing *C. glutamicum* *parA* gene fused with *cfp* | (Sieger & Bramkamp, 2015) |
| pCD10 | pETDuet containing *C. glutamicum* *parB* gene fused with *cfp* | (Donovan et al., 2012) |
| pJP108 | pBad/HisA- *icsA507-620-mcherry,* arabinose-inducible | (Ptacin et al., 2010) |
| pJP108*divIVA*_Ms_ | pJP108 containing *M. smegmatis* *divIVA* gene fused with *icsA507-620-mcherry* | (Pióro et al., 2019) |
| pACYC*egfp* | pACYCDuet-1 containing *egfp* gene | (Pióro et al., 2019) |
| pACYC*egfp-parA_Ms_* | pACYCDuet-1 containing *egfp* gene fused with *M. smegmatis* *parA* gene | (Pióro et al., 2019) |
| pACYC*egfp-parA_Cg_* | pACYCDuet-1 containing *egfp* gene fused with  *C. glutamicum* *parA* gene | This study |
| pACYC*egfp-parA_Cc_* | pACYCDuet-1 containing *egfp* gene fused with  *C. crescentus* *parA* gene | This study |
| pACYC*parB_Ms_-mneon* | pACYCDuet-1 containing *M. smegmatis parB* gene fused together with *mneon* | This study |
| pET28a*parB_Ms_-mneon* | pET28a kanamycin resistance vector, p_lac_ promoter, containing *M. smegmatis parB* gene fused together with *mneon* | This study |
| *parB_Cg_ -divIVA*_Ms_ | pET-Duet1, ampicillin resistance vector, containing *C. glutamicum* *parB* gene fused with *CFP* (MCS 1) and *M. smegmatis divIVA* gene fused with *mcherry* (MCS 2) | This study |
| *parB_Cg_-* *icsA* | pET-Duet1, ampicillin resistance vector, containing *C. glutamicum* *parB* gene fused with *CFP* (MCS 1) and *icsA* – *mcherry gene* (MCS 2) | This study |
| *parB_Cg_- divIVA*_Cg_ | pET-Duet1, ampicillin resistance vector, containing *C. glutamicum* *parB* gene fused with *CFP* (MCS 1) and *C. glutamicum divIVA* gene fused with *mcherry* (MCS 2) | This study |
| pETDuet*parB_Cc_-cfp* | pET-Duet1, ampicillin resistance vector, containing *C. crescentus* *parB* gene fused with *cfp* | This study |
| *parB_Cc_- divIVA*_Ms_ | pET-Duet1, ampicillin resistance vector, containing *C. crescentus* *parB* gene fused with *cfp* (MCS 1) and *M. smegmatis divIVA* gene fused with *mcherry* (MCS 2) | This study |
| *parB_Cc_-ics* | pET-Duet1, ampicillin resistance vector, containing *C. crescentus* *parB* gene fused with *cfp* (MCS 1) and *icsA* – *mcherry gene* (MCS 2) | This study |
| *parB_Cc_-divIVA*_Cg_ | pET-Duet1, ampicillin resistance vector, containing *C. crescentus* *parB* gene fused with *cfp* (MCS 1) and *C. glutamicum divIVA* gene fused with *mcherry* (MCS 2) | This study |
| **Constructs used for *M. smegmatis* modifications** | | |
| pMV306ø | kanamycin resistance, *oriE*, *oriM*, *attP*, *attB* integrative vector for mycobacterial transformation | (Triccas et al., 1998) |
| pMV306p_ami_*ø* | pMV306 containing acetamide inducible promoter p_ami_ | (Ginda et al., 2013) |
| pMV306p_ami_ *parA*_Ms_ | pMV306 containing *M. smegmatis parA* gene | (Ginda et al., 2013) |
| pMV306p_ami_ *parA*_Cc_ | pMV306 containing *C. crescentus parA* gene | This study |
| pMV306p_ami_ *parA*_Cg_ | pMV306 containing *C. glutamicum parA* gene | This study |
| **Constructs used for *C. crescentus* modifications** | | |
| pMT335 | Gentamycin resistance, p_van_ promoter inducible with vanillate | P. Viollier lab stock, Universite de Geneve, Geneva (Thanbichler et al., 2007) |
| pMT335*parA_Cc_* | pMT335 containing *C. crescentus parA* gene | This study |
| pMT335*parA_Ms_* | pMT335 containing *M. smegmatis parA* gene | This study |
| pMT335*parA_Rs_* | pMT335 containing *R. sphaeroides parA* gene | This study |
| **Constructs used for *C. glutamicum* modifications** | | |
| pEKEX2ø | E. *coli* - *C. glutamicum* shuttle vector for regulated gene expression (P*_tac_ lacI*^q^ pBL1 *oriV_C_*_._*_g_*_._ pUC18 *oriV_E_*_._*_c_*_._), kanamycin resistance | M. Bramkamp lab stock, Kiel University, Kiel (Donovan et al., 2010), (Eikmanns et al., 1991) |
| pEKEX2*parA_Cg_* | pEKEX2 containing *C. glutamicum parA* gene | This study |
| pEKEX2*parA_Ms_* | pEKEX2 containing *M. smegmatis parA* gene | This study |
| ***E. coli* strains** | | |
| DH5α | *F*-*, endA1, glnV44, thi*-*1, recA1, relA1, gyrA96, deoR,nupGΦ80dlacZΔM15Δ(lacZYA*-*argF) U169, hsdR17(rKmK+), λ*- | D. Jakimowicz lab stock, University of Wroclaw |
| BTH101 | F-, *cya-99*, *araD139, galE15, galK16, rpsL1 (Str ^r^)*, *hsdR2, mcrA1, mcrB1* | (Karimova et al., 1998) |
| BL21(DE3) | *F*-*, ompT, gal, dcm, lon, hsdSB(rB*- *mB*-*), λ(DE3)* | D. Jakimowicz lab stock, University of Wroclaw |
| ***M. smegmatis* strains** | | |
| WT | *M. smegmatis* mc^2^ 155 | D. Jakimowicz  lab stock, University of Wroclaw |
| KG22 | *M. smegmatis* mc^2^ 155 Δ*parA* | (Ginda et al., 2013) |
| KG11 | *M. smegmatis* mc^2^ 155 *attB*L5:: pMV306p_ami_ *parA*_Ms_ | (Ginda et al., 2013) |
| MP24 | *M. smegmatis* mc^2^ 155 *attB*L5:: pMV306p_ami_*ø* | This study |
| MP22 | *M. smegmatis* mc^2^ 155 *attB*L5:: pMV306p_ami_ *parA*_Cc_ | This study |
| MP28 | *M. smegmatis* mc^2^ 155 *attB*L5:: pMV306p_ami_ *parA*_Cg_ | This study |
| MP29 | *M. smegmatis* mc^2^ 155 Δ*parA attB*L5:: pMV306p_ami_ *parA*_Cg_ | This study |
| MP30 | *M. smegmatis* mc^2^ 155 Δ*parA attB*L5:: pMV306p_ami_ *ø* | This study |
| KG32 | *M. smegmatis* mc^2^ 155 Δ*parA attB*L5:: pMV306p_ami_ *parA*_Ms_ | K. Ginda unpublished, D. Jakimowicz lab stock, University of Wroclaw |
| KKOx49 | *M. smegmatis* mc^2^ 155 *parB-mneon* | (Trojanowski et al., 2017) |
| ***C. crescentus* strains**  This study | | |
| WT | *C. crescentus* WT NA 1000 | P. Viollier lab stock, University of Geneva |
| MT97 | *C. crescentus* WT NA 1000 *mipZ* :: *mipZ-yfp* | P. Viollier lab stock, University of Geneva (Thanbichler & Shapiro, 2006) |
| MP_Cc_1 | *C. crescentus* WT NA 1000 pMT335*ø* | This study |
| MP_Cc_2 | *C. crescentus* WT NA 1000 pMT335*parA_Ms_* | This study |
| MP_Cc_3 | *C. crescentus* WT NA 1000 pMT335*parA_Cc_* | This study |
| MP_Cc_4 | *C. crescentus* WT NA 1000 pMT335*parA_Rs_* | This study |
| MP_Cc_5 | *C. crescentus* WT NA 1000 *mipZ:*: *mipZ-yfp* pMT335ø | This study |
| MP_Cc_6 | *C. crescentus* WT NA 1000 *mipZ:*: *mipZ-yfp* pMT335*parA_Ms_* | This study |
| MP_Cc_7 | *C. crescentus* WT NA 1000 *mipZ:*: *mipZ-yfp* pMT335*parA_Cc_* | This study |
| MP_Cc_8 | *C. crescentus* WT NA 1000 *mipZ:*: *mipZ-yfp* pMT335*parA_Rs_* | This study |
| ***C. glutamicum* strains** | | |
| RES 167 | *Restriction deficient C. glutamicum* wild type strain | M. Bramkamp lab stock, Kiel University, Kiel |
| CDC001 | *C. glutamicum* Δ*parA* | (Donovan et al., 2010) |
| CDC008 | *C. glutamicum* Δ*parAparB-CFP* | (Donovan et al., 2010) |
| DJ_Cg1w | *C. glutamicum* WT, pEKEX2_*parA_Ms_* | This study |
| DJ_Cg2w | *C. glutamicum* Δ*parA*, pEKEX2_*parA_Ms_* | This study |
| DJ_Cg3w | *C. glutamicum* Δ*parAparB-ypet* pEKEX_*parA_Ms_* | This study |
| DJ_Cg4w | *C. glutamicum* WT, pEKEX2_*parA_Cg_* | This study |
| DJ_Cg5w | *C. glutamicum* Δ*parA*, pEKEX2_*parA_Cg_* | This study |
| DJ_Cg6w | *C. glutamicum* Δ*parAparB-ypet* pEKEX_*parA_Cg_* | This study |
| DJ_Cg7 | *C. glutamicum* WT, pEKEX2ø | This study |
| DJ_Cg8 | *C. glutamicum* Δ*parA*, pEKEX2ø | This study |
| DJ_Cg_9 | *C. glutamicum* Δ*parAparB-ypet* pEKEX2ø | This study |

**Table S2. Oligonucleotides used in this study**

| **Name** | **Sequence 5' to 3'** |
| --- | --- |
| ParACc_NcoIXbaFw | **CCATGG**GTCTAGAGATGTCCGCTAATCCTCTCCGC |
| ParACc_KpnIBamHIRv | G**GGTACC**G**GGATCC**TTAGGCGGCCTTGGCCT |
| ParBCc_XbaIBamHIFw | G**TCTAGA**G**GGATCC**ATGGAGTCCGTCGTGGTGGGA |
| ParBCc_EcoRIRv | C**GAATTC**TCAGATCCCGCGCGTCAGTCG |
| PopZ_SLIC_Rv | ACGGCCGAATTCTTAGTTACTTAGGTACCCGGGCGCCGCGTCCCCGA |
| PopZ_SLIC_Fw | GGTCGACTCTAGAGGATCCCCGGGTACCTAAATGTCCGATCAGTCTC |
| pKTmipZCcFw | GCTGCAGGGTCGACTCTAGAGATGGCCGAAACGCGC |
| pKTmipZCcRv | GTAAAACGACGGCCGAATTCACTGCGCCGCCAGC |
| ParACg_B2H_XbaIFw | CATTCTAGAGGAAGACACTACTTGGGAA |
| ParACg_B2HKpnIRv | CATGGTACCTTTCGCAGGTTTTAGGCC |
| ParAHybFw | C**GCTGAC**CCCATGGATACGCCGATCGCCGCGGAGGCCGAACAGG  CCACACGTGTCCTCCACAGCTCCATGGGATCTGACTCGAACCGAC |
| ParARsEcoRIXbaIRv | **TCTAGAGAATTC**TCATCCTCCTCCCAAGGCGGGT |
| ParACc_NcopACYCFw | G**CCATGG**CTATGTCCGCTAATCCTCTCCGC |
| ParACc_EcoRIRv | C**GAATTC**TTAGGCGGCCTTGGCCT |
| ParACg_XbaIFw | G**TCTAGA**ATGGAAGACACTACTTGGGAAGACACA |
| ParACg_KpnRv | C**GGTACC**CTATTTCGCAGGTTTTAGGC |
| ParBmNeonFw | G**CCATGG**CCATGAATCAGCCGGCACGCA |
| ParB_mNeon_Rv | G**GAATTC**TTATTTGTACAATTCATCCA |
| cherryFw_Nde | CC**CATATG**GTGAGCAAGGGCGAGGAGG |
| Div_revSnaBI | CC**TACGTA**TCAGTTGTTGCCGCGGTTGAACTGG |
| Ics_Fw_NdeI | **CATATG**AGTACTATTCTGGCAGATAATCTCAGCCATC |
| Cherry_rvSnaBI | CC**TACGTA**TTACTTGTACAGCTCGTCCATGCCGC |
| ParB_Cc_pET_Fw | C**GGATCC**GATGGAGTCCGTCGTGGTGGGAG |
| ParB_Cc_SalI_pET | C**GTCGAC**GATCCCGCGCGTCAGTCG |
| ParAMs_NdeFw | G**CATATG**GATACGCCGATCGCC |
| ParAMs_KpnIEcoRIRv | C**GGTACC**CG**GAATTC**CTACTGCTGGCGCGGCGGCGC |
| ParAMs_BamHI_Fw | CTCTAGAGG**GATCC**ATGGATACGCCGATCGCCGC |
| A1-pEX2-F | CAG**GGTACC**ATGGAAGACACTACTTGG |
| A1-pEX2-R | CAG**GAATTC**CTATTTCGCAGGTTTTAG |
| ParARs_NdeFw | G**CATATG**TCTGACTCGAACCGA |
| ParACc_NdeFw | G**CATATG**TCCGCTAATCCTCTCCGC |
| ParACc_EcoRIRv | C**GAATTC**TTAGGCGGCCTTGGCCT |

**Supplementary Materials and methods**

**Preparation of *E. coli* constructs for the BTH system**

The constructs for the BTH system and protein overproduction and purification were prepared as follows. The template for the *C. crescentus* constructs was *C. crescentus* WT NA 1000 chromosomal DNA. For PCR amplification of the *C. crescentus* *parA* gene, ParACc_NcoIXbaFw and ParACcKpnIBamHIRv primers were used. The PCR product and vectors pUT18C and pKT25 were cut by XbaI and BamHI restriction enzymes and ligated. The *C. crescentus* *parB* gene was amplified with ParBCc_XbaIBamHIFw and ParBCc_EcoRIRv primers, digested together with pUT18C and pKT25 with XbaI and EcoRI restriction enzymes and ligated. The *C. crescentus* *popZ* and *mipZ* genes were cloned into pKT25 using the SLIC (sequence ligation independent cloning) method (Li & Elledge, 2012). First, the *popZ* and *mipZ* genes were amplified with the PopZ_SLIC_Rv and PopZ_SLIC_Fw primer pairs and the pKTmipZCcFw and pKTmipZCcRv primer pairs, respectively. pKT25 was digested with XbaI and KpnI enzymes and used for SLIC of both *mipZ* and *popZ* gene PCR products to deliver pKT25*mipZ* and pKT25*popZ* vectors*.* pUT18C*mipZ* and pUT18C*popZ* were created by restriction digestion of pKT25*mipZ* and pKT25*popZ*, respectively, with the XbaI and KpnI enzymes and ligation of the obtained *mipZ* and *popZ* genes with pUT18C cleaved with the same enzymes.

Chromosomal DNA of *C. glutamicum* RES 167 served as template for parA_Cg_ BTH constructs. Primer pair ParACg_B2H_XbaIFw/ ParACg_B2HKpnIRv was used for amplification of parA_Cg_. The resulting PCR product and plasmids pKT25/ pUT18C were digested using KpnI and XbaI and ligated subsequently, resulting in pKT25*parA*_Cg_ and pUT18C*parA*_Cg_.

For preparation of the pKT25*hybrid_parA* construct, the *hybrid_parA* gene (N-terminal extended *R. sphaeroides parA)* was first amplified (pKT25*parA*_Rs_ was used as a template) with the ParAHybFw and ParARsEcoRIXbaIRv primers, digested by SalI and EcoRI and ligated to the pKT25 vector digested with the same enzymes. The elongated ParAHybFw primer codes the first 20 amino acids of *M. smegmatis* ParA, and the rest of the primer is complementary to the beginning of the *R. sphaeroides parA* gene. The ligation mixtures were used for transformation of DH5α cells. The obtained clones were verified using PCR, enzyme digestion and sequencing.

**Constructs for *E. coli* colocalisation**

pACYCDuet*egfp-parA_Cc_* was constructed using the *C. crescentus parA* gene amplified with ParACc_NcopACYCFw and ParAC.c._EcoRIRv primers and the template of pET21a*parA_Cc_*. PCR product and pACYCDuet*egfp* were digested with NcoI and EcoRI restriction enzymes and ligated.

pACYCDuet*egfp-parA_Cg_* was constructed using the *Corynebacterium glutamicum* *parA* gene excised from pCD17 plasmid using SacI and partial NcoI digestion and cloned into the pACYCDuet*egfp-parA_Ms_* vector cleaved with the same enzymes.

The pET28a*parB_Ms_-mneon* vector was constructed as follows. For amplification of the *M. smegmatis* *parB* gene, ParBmNeonFw and ParB_mNeon_Rv primers and the *M. smegmatis* KKox49 chromosomal DNA as the template were used. The PCR product and pET28a vector were digested with NcoI and EcoRI restriction enzymes and ligated.

The pACYC*parB_Ms_-mneon* vector was constructed as follows. The pACYC-Duet1 vector was cut with NcoI and EcoRI, and the *parB_Ms_-mneon* gene was excised from the pET28a*parB_Ms_-mneon* vector with NcoI and EcoRI and ligated.

pETDuet*parB_Cc_*-*cfp* was constructed as follows. The *C. crescentus* *parB* gene was amplified using the ParB_Cc_pET_Fw and ParB_Cc_SalI_pET primers, using *C. crescentus* WT NA 1000 chromosomal DNA as a template. The PCR product and pETDuet*cfp* vector were digested with BamHI and SalI restriction enzymes and ligated.

To obtain pETDuet derivatives with *parB_Cg_-cfp* and *divIVA_Cg_-mcherry* or *parB_Cc_-cfp* and *divIVA_Cg_-mcherry,* the *divIVA*_Cg-_*mcherry* gene was cut from pCD74 (pETDuet*divIVA_Cg_-mcherry*) with NdeI and PacI enzymes and cloned into pETDuet*parB_Cc_*-*cfp* as well as CD10 (pETDuet*parB_Cg_-cfp*), delivering *parB_Cc_-divIVA*_Cg_ and *parB_Cg_-divIVA*_Cg_.

To obtain pETDuet derivatives with *parB_Cg_-cfp* and *divIVA_Ms_-mcherry* and *parB_Cc_-cfp* and *divIVA_Ms_-mcherry,* vectors pETDuet*parB_Cc_-cfp* and pCD10 (pETDuet*parB_Cg_-cfp*) were digested with EcoRV and NdeI, and the *divIVA_Ms_-mcherry* gene was PCR amplified with pJP108divIVA_Ms_ as a template and cherry_Fw_Nde and div_revSnaBI primers. The PCR product was digested with NdeI and SnaBI enzymes and ligated into vectors digested with NdeI and EcoRV, yielding *parB_Cc_-divIVA*_Ms_ and *parB_Cg_-divIVA*_Ms_.

To construct pETDuet derivatives containing *parB_Cg_-cfp* and *icsA-mcherry* or *parB_Cc_-cfp* and *icsA-mcherry*, vectors pETDuet*parB_Cc_-cfp* and pCD10 (pETDuet*parB_Cg_-cfp*) were digested with EcoRV and NdeI, and the *icsA-mcherry* gene was PCR amplified with pJP108*divIVA_M_*_s_ as a template and Ics_Fw_NdeI and cherry_rvSnaBI primers. The PCR product was digested with NdeI and SnaBI enzymes and ligated into vectors digested with NdeI and EcoRV, yielding *parB_Cg_-icsA and parB_Cc_-icsA*.

All the obtained constructs were analysed by PCR, restrictive digestion and sequencing when PCR amplification was involved.

**Preparation of *E. coli* constructs for protein overproduction and purification**

To construct pGEX6P2*parB_Cc_*, the *C. crescentus* *parB* gene was amplified with ParBCc_XbaIBamHIFw and ParBCc_EcoRIRv primers, and *C. crescentus* WT NA 1000 chromosomal DNA was used as the template. Next, the PCR product was digested with BamHI and EcoRI together with pGEX6P2 and ligated. The pET28*parA*_cc_ construct was prepared by excision of the *parA* gene from the pET21a*parA*_Cc_ construct with the enzymes NdeI and HindIII and ligated into the pET28a vector cut with the same enzymes.

**Protein purification**

The *C. crescentus* ParA, *C. glutamicum* ParA and ParB proteins were purified as N-terminal His-tagged proteins using nickel-affinity chromatography. Briefly, cells from a 1.6 litre culture were lysed by sonication in 50 mL of PAA buffer (100 mM HEPES/KOH [pH 7.4], 450 mM KCl, 2 mM MgCl_2_, 50 mM potassium glutamate, 1 mM DTT), and the cellular extract was clarified by centrifugation (25,000 g, 45 min, 4 °C). Next, proteins were precipitated with ammonium sulfate (40% saturation), resuspended in 2.5 mL of PAA buffer and applied to a PD-10 desalting column. Proteins were eluted with 3.5 mL of PAB buffer (PAA buffer supplemented with 10 mM imidazole and 0.1 mM ADP), diluted to 50 mL with PAB buffer and incubated (4 °C, 16 h) with a 1 mL bed volume of Ni-NTA agarose resin (Qiagen). The resin was washed three times with PAB buffer, and bound proteins were eluted with PAC buffer (buffer PAA supplemented with 500 mM imidazole and 10% glycerol).

The *M. smegmatis* ParB protein was purified as a C-terminal His-tagged fusion protein using nickel-affinity chromatography. Briefly, cells from a 0.8 litre culture were lysed by sonication in 50 mL of PBS supplemented with 1 mM imidazole, and the cellular extract was clarified by centrifugation (25,000 g, 45 min, 4 °C). Clarified lysate was incubated (4 °C, 16 h) with a 1 mL bed volume of Ni-NTA agarose resin (Qiagen). The resin was washed three times with lysis buffer, and bound proteins were eluted with PBS supplemented with 500 mM imidazole.

The *C. crescentus* ParB and *M. smegmatis* ParA proteins were purified using a GST-fusion protein purification system (GE Healthcare) according to the manufacturer’s protocol. Briefly, cells from a 1.6 litre culture were lysed by sonication in 50 mL of buffer PGA (50 mM Tris-HCl [pH 8.0], 1 mM DTT and 10% glycerol), and the cellular extract was clarified by centrifugation (25,000 g, 45 min, 4 °C). Clarified lysate was incubated (4 °C, 16 h) with a 0.5 mL bed volume of GSH-Sepharose (GE Healthcare). The resin was washed two times with 10 mL PGA buffer and one time with 10 mL PreS buffer (50 mM Tris-HCl, 150 mM NaCl, 1 mM DTT, 1 mM EDTA, pH 7.5). Resin-bound proteins were subjected to PreScission protease treatment overnight, according to the manufacturer’s protocol (GE Healthcare). The resin was centrifuged (500 g, 5 min, 4 °C), and the supernatant (containing proteins of interest) was collected.

***M. smegmatis* strain construction**

All vectors for *M. smegmatis* transformations were prepared in *E. coli* DH5α. To construct pMV306p_ami_ derivatives, the *C. crescentus* *parA* gene was PCR amplified with the ParACc_NcoIXbaFw and ParACc_KpnIBamHIRv primers and pKT25*parA*_Cc_ as a template, while the *C. glutamicum* *parA* gene was amplified with the ParACg_XbaIFw and ParACg_KpnRv primers and pKT25*parA*_Cg_ as a template. PCR products as well as pMV306p_ami_ were digested with XbaI and KpnI restriction enzymes and ligated to yield pMV306p_ami_*parA*_Cc_ and pMV306p_ami_*parA*_Cg_*.* The obtained constructs were verified by PCR, restriction cloning and sequencing.

Next, the obtained constructs, as well as the control vector pMV306p_ami_, were used to transform wild-type and *parA* deletion *M. smegmatis* (KG22) strains using the standard protocol (Triccas et al., 1998). Transformants were selected on medium containing kanamycin (50 μg/mL) and verified by PCR.

**Construction of *C. glutamicum* strains**

To construct *C. glutamicum* strains producing *C. glutamicum* ParA and *M. smegmatis* ParA, pEKEX2 derivatives were constructed by inserting *parA* genes under the p_lac_ promoter. To this end, the *M. smegmatis parA* gene was amplified with the ParAMs_BamHI_Fw and ParAMs_KpnIEcoRIRv primers, digested with the BamHI and EcoRI restriction enzymes and cloned into the pEKEX-2 enzyme vector. *C. glutamicum* *parA* was amplified using A1-pEX2-F and A1-pEX2-R primers digested with EcoRI and KpnI and cloned into pEKEX-2 digested with the same enzymes. The obtained pEKEX2*parA_Ms_* and pEKEX2*parA*_Cg_ were used for transformation of the *C. glutamicum* wild-type and *parA* deletion strains, and transformants were selected on media supplemented with kanamycin (25 μg/mL).

**Construction of *C. crescentus* strains**

To prepare constructs for *C. crescentus* strain analysis, derivatives of pMT335 vectors were prepared in *E. coli*. First, *parA* genes were amplified using specific primers: *M. smegmatis* *parA* with ParAMs_NdeFw, ParAMs_KpnIEcoRIRv, *C. crescentus* *parA* with ParACc_NdeFw, ParACc_EcoRIRv and *R. sphaeroides* *parA* with ParARs_NdeFw and ParARsEcoRIXbaIRv, using pKT25 containing appropriate *parA* genes as the templates. Next, the obtained PCR products and pMT335 vector were digested with restriction enzymes NdeI and EcoRI and ligated. The obtained pMT335*parA_Cc_*, pMT335*parA_Ms_*, and pMT335*parA_Rs_* constructs were verified by sequencing.

The obtained pMT335 derivatives were used for transformation of *C. crescentus* wild-type and MT97 strains. Transformants were selected on PYE medium (peptone-yeast extract) containing gentamycin (1 µg/mL) and verified by PCR.

**References**

Corrales-Guerrero, L., He, B., Refes, Y., Panis, G., Bange, G., Viollier, P. H., Steinchen, W., & Thanbichler, M. (2020). Molecular architecture of the DNA-binding sites of the P-loop ATPases MipZ and ParA from Caulobacter crescentus. *Nucleic Acids Research*, *48*(9), 4769–4779. https://doi.org/10.1093/nar/gkaa192

Donovan, C., Schwaiger, A., Krämer, R., & Bramkamp, M. (2010). Subcellular Localization and Characterization of the ParAB System from Corynebacterium glutamicum. *Journal of Bacteriology*, *192*(13), 3441–3451. https://doi.org/10.1128/JB.00214-10

Donovan, C., Sieger, B., Krämer, R., & Bramkamp, M. (2012). A synthetic Escherichia coli system identifies a conserved origin tethering factor in Actinobacteria. *Molecular Microbiology*, *84*(1), 105–116. https://doi.org/10.1111/j.1365-2958.2012.08011.x

Eikmanns, B. J., Kleinertz, E., Liebl, W., & Sahm, H. (1991). A family of Corynebacterium glutamicum/Escherichia coli shuttle vectors for cloning, controlled gene expression, and promoter probing. *Gene*, *102*(1), 93–98. https://doi.org/10.1016/0378-1119(91)90545-m

Ginda, K., Bezulska, M., Ziólkiewicz, M., Dziadek, J., Zakrzewska-Czerwińska, J., & Jakimowicz, D. (2013). ParA of Mycobacterium smegmatis co-ordinates chromosome segregation with the cell cycle and interacts with the polar growth determinant DivIVA. *Molecular Microbiology*, *87*(5). https://doi.org/10.1111/mmi.12146

Jakimowicz, D., Brzostek, A., Rumijowska-Galewicz, A., Żydek, P., Dołzbłasz, A., Smulczyk-Krawczyszyn, A., Zimniak, T., Wojtasz, Ł., Zawilak-Pawlik, A., Kois, A., Dziadek, J., & Zakrzewska-Czerwińska, J. (2007). Characterization of the mycobacterial chromosome segregation protein ParB and identification of its target in Mycobacterium smegmatis. *Microbiology (Reading, England)*, *153*(Pt 12), 4050–4060. https://doi.org/10.1099/mic.0.2007/011619-0

Karimova, G., Pidoux, J., Ullmann, A., & Ladant, D. (1998). A bacterial two-hybrid system based on a reconstituted signal transduction pathway. *Proceedings of the National Academy of Sciences*, *95*(10), 5752–5756. https://doi.org/10.1073/pnas.95.10.5752

Li, M. Z., & Elledge, S. J. (2012). SLIC: A method for sequence- and ligation-independent cloning. *Methods in Molecular Biology (Clifton, N.J.)*, *852*, 51–59. https://doi.org/10.1007/978-1-61779-564-0_5

Pióro, M., Małecki, T., Portas, M., Magierowska, I., Trojanowski, D., Sherratt, D., Zakrzewska-Czerwińska, J., Ginda, K., & Jakimowicz, D. (2019). Competition between DivIVA and the nucleoid for ParA binding promotes segrosome separation and modulates mycobacterial cell elongation. *Molecular Microbiology*, *111*(1), 204–220. https://doi.org/10.1111/mmi.14149

Ptacin, J. L., Lee, S. F., Garner, E. C., Toro, E., Eckart, M., Comolli, L. R., Moerner, W. E., & Shapiro, L. (2010). A spindle-like apparatus guides bacterial chromosome segregation. *Nature Cell Biology*, *12*(8), 791–798. https://doi.org/10.1038/ncb2083

Sieger, B., & Bramkamp, M. (2015). Interaction sites of DivIVA and RodA from Corynebacterium glutamicum. *Frontiers in Microbiology*, *5*, 738. https://doi.org/10.3389/fmicb.2014.00738

Sieger, B., Schubert, K., Donovan, C., & Bramkamp, M. (2013). The lipid II flippase RodA determines morphology and growth in Corynebacterium glutamicum. *Molecular Microbiology*, *90*(5), 966–982. https://doi.org/10.1111/mmi.12411

Thanbichler, M., Iniesta, A. A., & Shapiro, L. (2007). A comprehensive set of plasmids for vanillate- and xylose-inducible gene expression in Caulobacter crescentus. *Nucleic Acids Research*, *35*(20), e137. https://doi.org/10.1093/nar/gkm818

Thanbichler, M., & Shapiro, L. (2006). MipZ, a spatial regulator coordinating chromosome segregation with cell division in Caulobacter. *Cell*, *126*(1), 147–162. https://doi.org/10.1016/j.cell.2006.05.038

Triccas, J., Parish, T., Britton, W., & Gicquel, B. (1998). An inducible expression system permitting the efficient purification of a recombinant antigen from Mycobacterium smegmatis. *EMS Microbiol Lett*, *167*, 151–156.

Trojanowski, D., Hołówka, J., Ginda, K., Jakimowicz, D., & Zakrzewska-Czerwińska, J. (2017). Multifork chromosome replication in slow-growing bacteria. *Scientific Reports*, *7*, 43836. https://doi.org/10.1038/srep43836
